# Emergence of a novel sublineage, MYMBD21 under SA-2018 lineage of Foot-and-Mouth Disease Virus serotype O in Bangladesh

**DOI:** 10.1038/s41598-023-36830-w

**Published:** 2023-06-17

**Authors:** Kazi Alamgir Hossain, Humaira Anjume, K. M. Mazharul Alam, Ashabul Yeamin, Salma Akter, M. Anwar Hossain, Munawar Sultana

**Affiliations:** 1 Department of Microbiology, University of Dhaka, Dhaka, 1000 Bangladesh; 2 Present Address: Department of Mathematics and Natural Sciences, BRAC University, Dhaka, Bangladesh; 3 Present Address: Department of Microbiology, Jahangirnagar University, Savar, Dhaka, 1342 Bangladesh; 4 Present Address: Jashore University of Science and Technology, Jashore, 7408 Bangladesh

**Keywords:** Microbiology, Virology

## Abstract

Foot-and-Mouth Disease (FMD) hinders the growth of the livestock industry in endemic countries like Bangladesh. The management and prevention of FMD are severely impacted by the high mutation rate and subsequent frequent generation of newer genotypes of the causative agent, Foot-and-Mouth Disease Virus (FMDV). The current study was conducted in nine districts of Bangladesh during 2019–21 to characterize the circulating FMDV strains based on the VP1 sequence analysis, the major antigenic recognition site providing serotype specificity and high variability of FMDV. This study detected the first emergence of the SA-2018 lineage in Bangladesh along with the predominance of Ind-2001e (or Ind-2001BD1) sublineage of ME-SA topotype under serotype O during 2019–21. The mutational spectrum, evolutionary divergence analysis and multidimensional plotting confirmed the isolates collected from Mymensingh districts, designated as MYMBD21 as a novel sublineage under the SA-2018 lineage. Analysis of the amino acid sequence revealed several changes in the G-H loop, B-C loop and C-terminal region of VP1, revealing a 12–13% divergence from the existing vaccine strains and a 95% VP1 protein homology, with most of the mutations potentially considerable as vaccine escape mutations, evidenced by three-dimensional structural analysis. This is the first report on the emergence of the SA-2018 lineage of ME-SA topotype of FMDV serotype O in Bangladesh, as well as a possible mutational trend towards the emergence of a distinct sublineage under SA-2018 lineage, which calls for in-depth genome-wide analysis and monitoring of the FMD situation in the country to implement a strategic vaccination and effective FMD control program.

## Introduction

Foot-and-Mouth Disease (FMD) is an acute and highly contagious disease that affects cloven-hoofed animals like cattle, pigs, sheep, goats, and water buffalo^1^. FMD is widespread in many areas around the globe, especially in Africa and Asia including Bangladesh. The causative agent is a notorious virus called Foot-and-Mouth Disease Virus (FMDV) which belongs to the genus *Aphthovirus* and the family *Picornaviridae*^2–4^. The FMDV possesses a positive-sense single-stranded RNA genome of about 8.5 kb surrounded by an icosahedral capsid with 60 copies of each of four structural proteins VP1, VP2, VP3, and VP4^4^. Among the capsid proteins, VP1 capsid protein possesses three out of five major neutralizing antigenic sites: antigenic sites 1, 3, and 5^5^. VP1 sequence data analyses are widely utilized for molecular characterization of field FMDV strains and revealing evolutionary divergence among individual virus strains^3,6–10^.

In Bangladesh, serotype O, A and Asia1 were found to circulate among the 7 distinct serotypes of FMDV (Euro-Asiatic serotypes A, O, C, and Asia1 and Southern African Territories [SAT] serotypes SAT1, SAT2, and SAT3)^2,11–13^. Among them, serotype O is the most predominant one and was responsible for 82% of the outbreaks in Bangladesh during 2012–2016^3^. Serotype O can be subdivided into 11 topotypes but only the Middle East-South Asia (ME-SA) topotype is found in Bangladesh. So far, there are four established lineages under the ME-SA topotype, namely Ind-2001, Ind-2011, PanAsia and PanAsia-2^3,14,15^. But two other endemic lineages, Pak-98 from Pakistan and Srl-97 from Sri Lanka were also documented. These lineages (Pak-98 and Srl-97) were endemic and were not detected outside the respective countries^16,17^. Ind-2001 lineage is diversified into five sublineages: Ind-2001a, b, c, d, and e^3,6,18^. Recently, a distinct novel lineage, SA-2018 under ME-SA topotype was reported from India in 2018^19^. The high mutation rate of the viral genome, lack of proofreading activity of the RNA polymerase and the quasi-species nature of the virus are responsible for the emergence of genetically and antigenically diverse strains of FMDV^19,20^.

In Bangladesh, Ind-2001d, Ind-2001BD1 and Ind-2001BD2 under the Ind-2001 lineage of ME-SA topotype were found to circulate^3^. Later, Ind-2001BD1 was designated as Ind2001e by the World Reference Laboratory for Foot-and-Mouth Disease (WRLFMD)^6,19^. The Ind-2001e or Ind-2001BD1 sublineage was predominant over the other sublineages^3^. Ind-2001BD2 sublineage was confirmed as a novel sublineage in a previous study and circulation of this particular sublineage was not found after 2013. Few uncategorized isolates (BD_BAU_ML1_2013; BD_BAU_ML2_2013; BD_SI_5_2013; O/BAN/BLRI/450.2/2018) were reported during 2013–18^21^. BD_BAU_ML1_2013 and BD_BAU_ML2_2013 isolates were reported as PanAsia-2 lineage^22^ as the isolates shared 98–99% identity with some Indian isolates reported in 2011 but these sequences were not genetically characterized^23^. Therefore, no clear information was provided about the lineage of these two isolates.

The existence of immunogenically diverse strains of FMDV without cross-protective immunity contributes to the devastating outbreaks each year in Bangladesh. FMD outbreaks appear as a threat to the economy of Bangladesh. To prevent the disease realistically, a well-planned control strategy must be designed that requires constant surveillance of the circulating FMDV strains and measuring the effectiveness of the existing vaccines against the circulating local strains. This study focused on molecular typing of the FMDV serotype O isolates collected during 2019–21 and reported the infiltration of a new lineage from the neighboring country as well as a mutational trend towards the emergence of a novel sublineage under the recently introduced lineage in Bangladesh. Antigenic heterogeneity of the newly emerged strain against existing vaccine strains was also documented in this study.

## Results

### Identification of serotypes: serotype O is prevalent in Bangladesh

**Figure 1:**
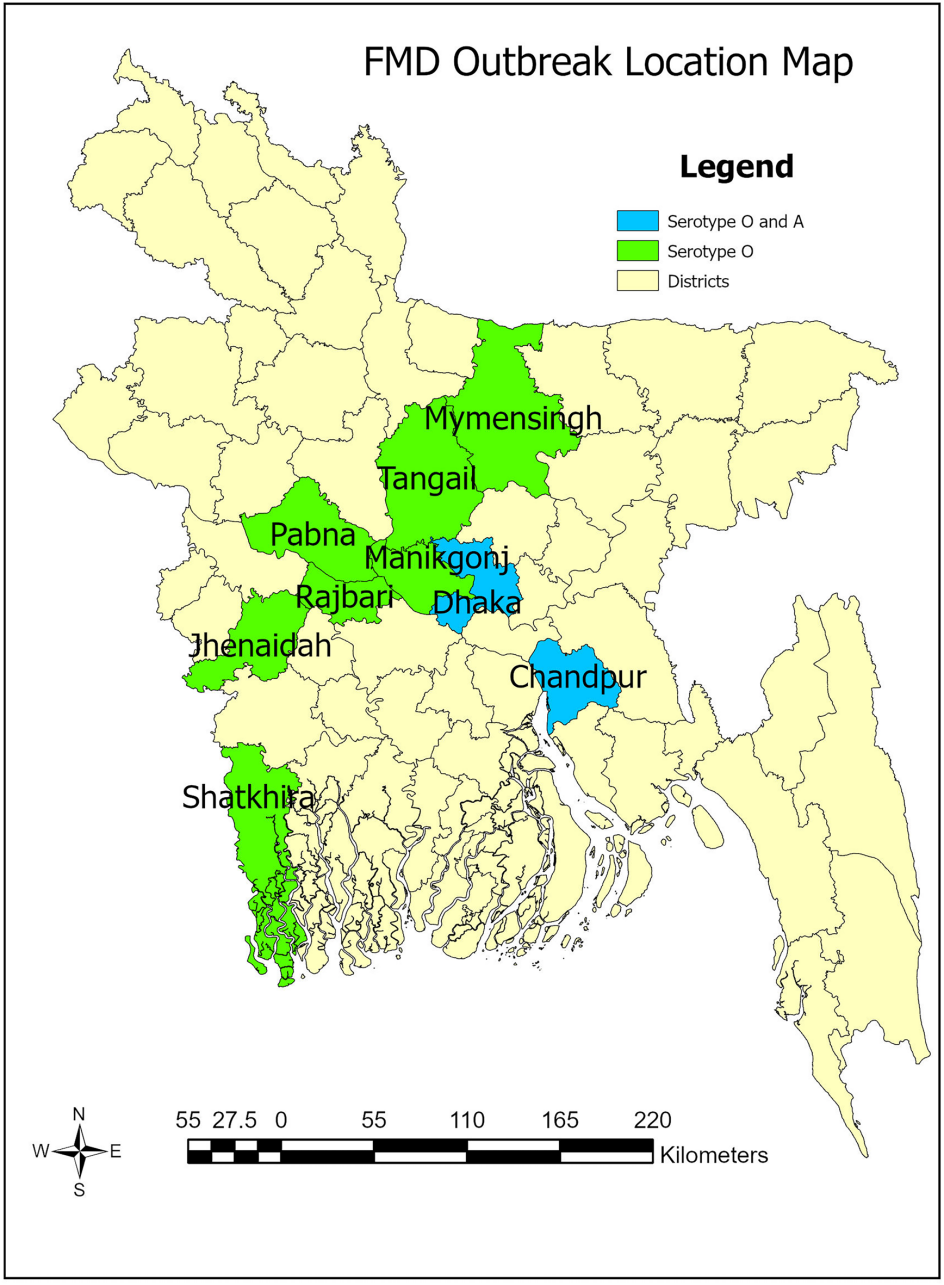
Geographical distribution of FMD outbreaks caused by FMDV serotype O and A in Bangladesh during 2019–2021. The map was generated using ArcGIS Pro 3.1.0 (https://www.esri.com/en-us/arcgis/products/arcgis-pro/overview)^41^ using data from the DIVA-GIS website (www.diva-gis.org).

A total of 147 representative epithelial tissue samples from nine districts of Bangladesh were selected (out of 156) for PCR-based diagnostic assay covering 24 suspected clinical outbreaks of FMD during 2019 to 2021. VP1 region based PCR assay detected 105 samples positive for FMDV. Among the positive samples, the VP1 region of 46 samples covering each outbreak area were sequenced and submitted to the NCBI GenBank database (August, 2022). Accession numbers of the sequences can be found in Supplementary Table S6. VP1 sequence data analysis by NCBI BLAST search revealed that out of 105 VP1 positive samples, 98 samples in 22 (91.3%) outbreaks were identified as serotype O, while 7 samples in 2 (8.7%) outbreak areas were found positive for serotype A from 2019 to 2021 in Bangladesh. In 2021, all of the outbreaks were due to the FMVD serotype O. Serotype O was found as the dominant serotype over serotype A and no serotype Asia1 case was detected in this study. In Fig. 1, the geographical locations of outbreaks and associated serotypes of FMDV were indicated. This study is focused on the subtyping of the serotype O isolates (n = 41).

### Phylogenetic analysis

**Figure 2:**
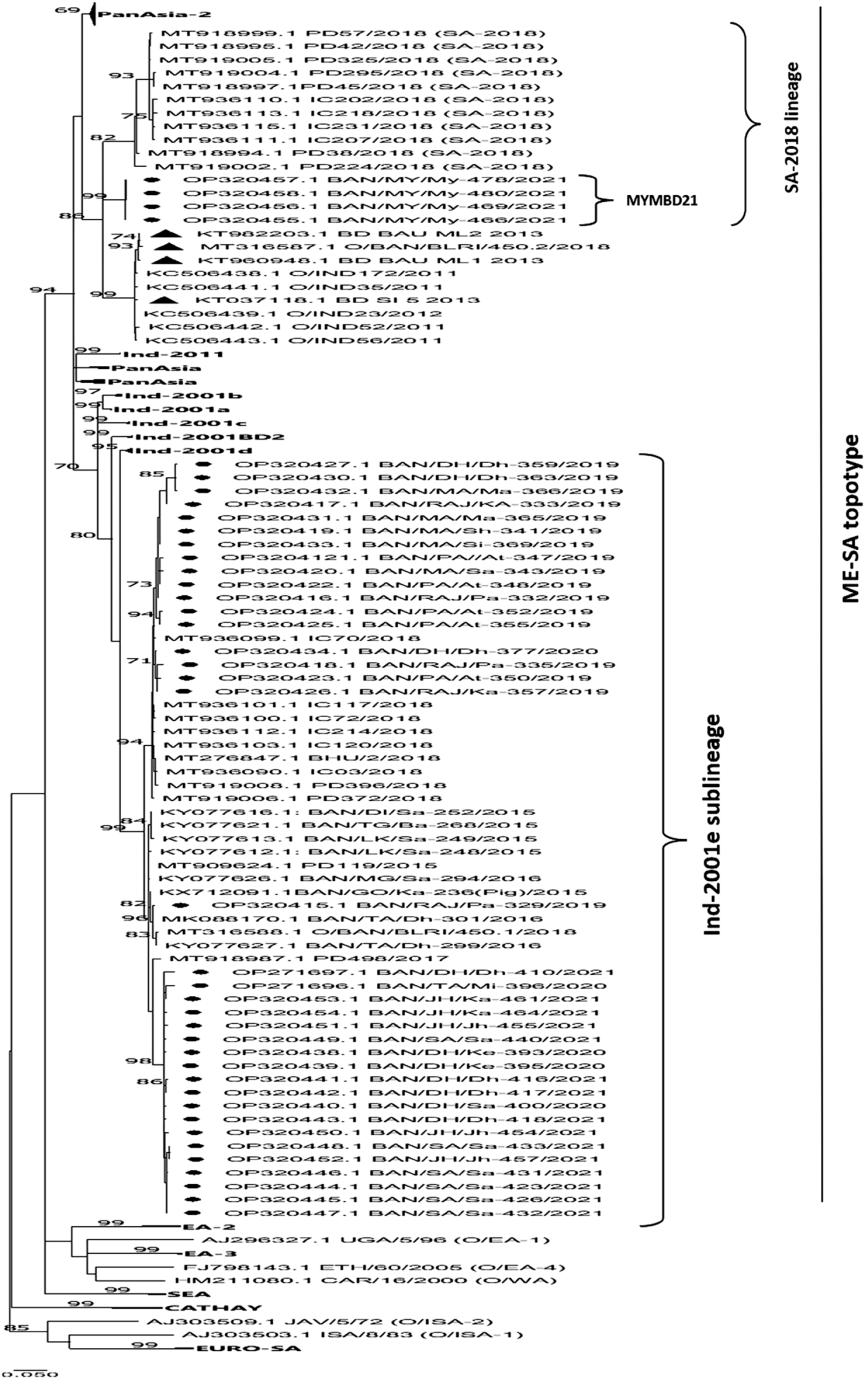
Phylogenetic reconstruction based on Maximum-Likelihood method using Tamura-Nei method in MEGA11^28^ of circulating serotype O in Bangladesh during 2019–2021. Sample sequences of this study are marked with ●. Uncharacterized Bangladeshi isolates are marked with ▲. Sequences belonging to the same clade are compressed for better visualization of the phylogenetic relationship.

Phylogenetic reconstruction based on the Maximum Likelihood method including serotype O sequences revealed that 37 out of 41 sequences clustered within the Ind-2001 lineage of the Middle East-South Asia (ME-SA) topotype. A distinct cluster was formed by 4 representative sequences of the Mymensingh district showing closeness to a recently reported lineage, called, SA-2018 from India^19^ and previous uncategorized isolates from Bangladesh^21,22^ under the same ME-SA topotype (Fig. 2). The isolates of the Ind-2001 lineage that circulated during 2019–21 (n = 37) mostly belonged to the Ind-2001e (or Ind-2001BD1) sublineage. From NCBI-BLAST search, it was revealed that the sequences from 2019 isolates and a single sequence from 2020 isolates (only BAN/DH/Dh-377/2020 isolate) had 98–99% identity with Indian isolates and 97% with Bangladeshi isolates under the Ind-2001e sublineage whereas rest of the 2020–21 isolates shared 97% identity with both Bangladeshi and Indian isolates of Ind-2001BD1 or Ind-2001e sublineage (Supplementary Table S1). It is noteworthy that the genetic distance calculated between Ind-2001BD1 and Ind-2001e in MEGA11 using the Kimura-2 parameter model was 0.026 which is less than the lowest genetic distance (0.044) calculated among established sub-lineages of Ind-2001 lineage which confirms that Ind-2001BD1 and Ind-2001e are the same sub-lineage (Supplementary Table S1, Table S2). The sequences from the Mymensingh district, named MYMBD21 in this study showed an evolutionary relationship to SA-2018 isolates, reported from India in 2018 (Fig. 2). MYMBD21 formed a completely distinct clade from the Indian isolates of the SA-2018 lineage with 5–6% divergence (Supplementary Fig. S1, Fig. S2, Fig. S3). MYMBD21 also showed a relationship with previous Bangladeshi strains which were reported during 2013 (named as PanAsia-2 lineage) and 2018 (uncategorized)^21,22^ but these isolates were 8% distant from both the MYMBD21 and Indian isolates of SA-2018 (Supplementary Fig. S4). The genetic identity of MYMBD21 was closer to SA-2018 than previous unclassified isolates of Bangladesh (Table 1; Supplementary Table S3).

**Table 1:** Estimates of evolutionary divergence over sequence pairs between lineages.

Lineage 1	Lineage 2	Genetic distance	Standard error
PanAsia	PanAsia-2	0.102	0.010
SA-2018	PanAsia	0.129	0.015
SA-2018	PanAsia-2	0.124	0.014
SA-2018	Previously reported Bangladeshi isolates (Uncategorized)	0.101	0.014
Previously reported Bangladeshi isolates (Uncategorized)	PanAsia	0.110	0.014
Previously reported Bangladeshi isolates (Uncategorized)	PanAsia-2	0.119	0.014
MYMBD21	PanAsia	0.108	0.014
MYMBD21	PanAsia-2	0.105	0.012
MYMBD21	SA-2018	0.062	0.010
MYMBD21	Previously reported Bangladeshi isolates (Uncategorized)	0.089	0.014

### Evolutionary divergence analysis

Evolutionary divergence between established lineages (PanAsia, PanAsia-2, SA-2018) of serotype O was estimated including MYMBD21. MYMBD21 isolates showed a genetic distance of 0.062 with SA-2018 which is lower than the genetic distance between two distinct lineages confirming that MYMBD21 falls within SA-2018 lineage and the nucleotide divergence of almost 5–6% further suggests MYMBD21 isolates belong to a distinct sublineage under SA-2018 lineage (Table 1; Supplementary Table S3).

On the other hand, the genetic distances of previously circulating uncharacterized Bangladeshi isolates (BD_BAU_ML1_2013, BD_BAU_ML2_2013, BD_SI_5_2013, O/BAN/BLRI/450.2/2018) with SA-2018 lineage and MYMBD21 were 0.101 and 0.089, respectively that was almost similar to distances among established lineages indicating existence of the SA-2018 as a separate lineage from these uncharacterized Bangladeshi isolates and the first occurrence of SA-2018 lineage in Bangladesh (Table 1; Supplementary Table S3).

### Multidimensional scaling

**Figure 3:**
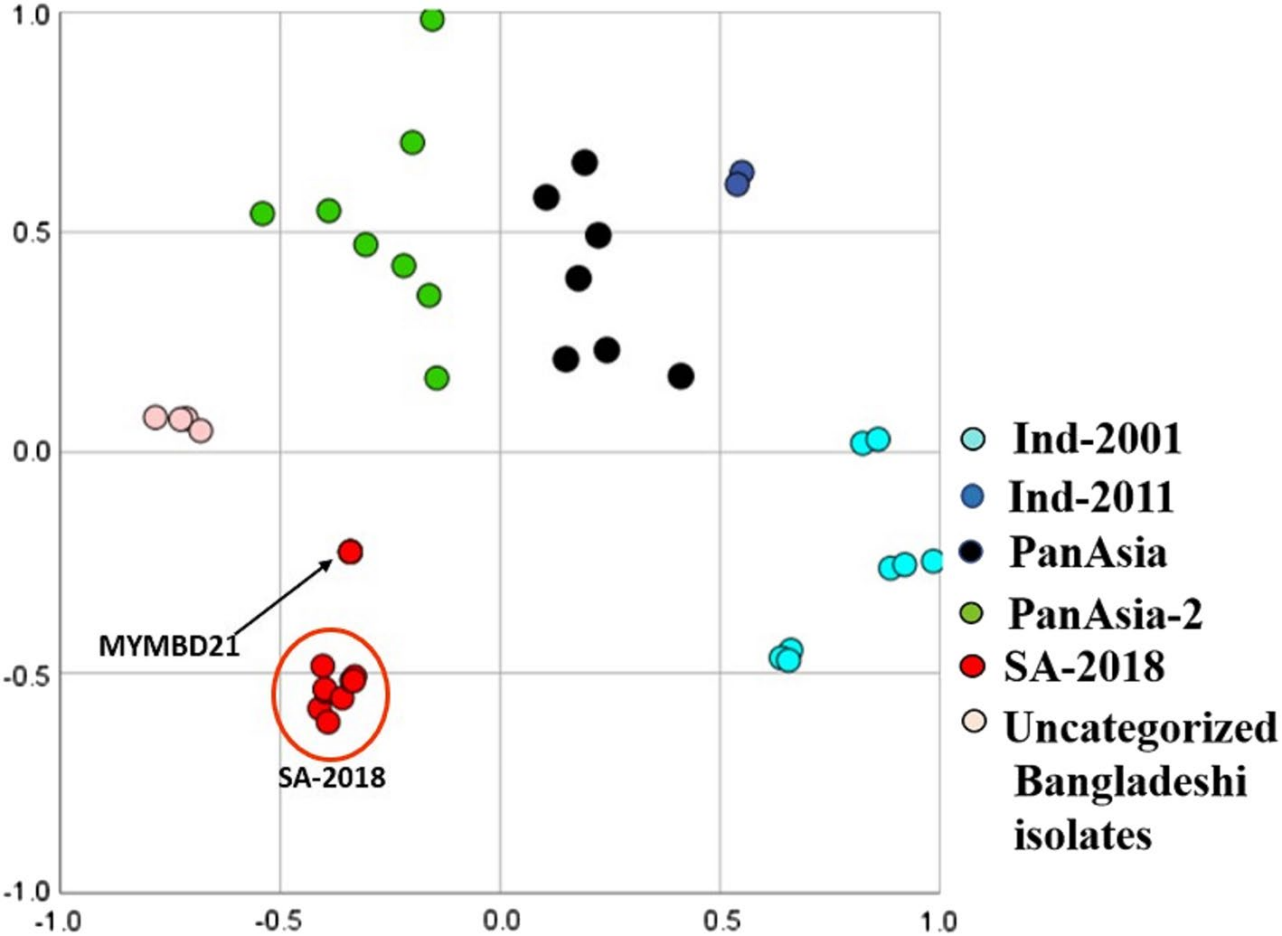
Multidimensional scaling plot. The plot is showing cluster formation by established lineages and sublineages of FMDV serotype O sequences along with newly emerged MYMBD21 sublineage in two-dimensional space based on the pairwise genetic distances calculated using the Kimura-2 parameter model. Uncategorized Bangladeshi samples and other lineages are clustered separately in the plot. The plot is generated using IBM SPSS Statistics (Version 26).

From the Multi-Dimensional Scaling (MDS) plot, it was illustrated that MYMBD21 isolates formed a completely separate cluster from other lineages of serotype O (Fig. 3). But the distance of the MYMBD21 cluster was the lowest with SA-2018 cluster compared to all other clusters formed by each lineage or sublineage.

### Mutational analysis

#### Comparative mutational analysis between MYMBD21 and SA-2018

**Figure 4:**
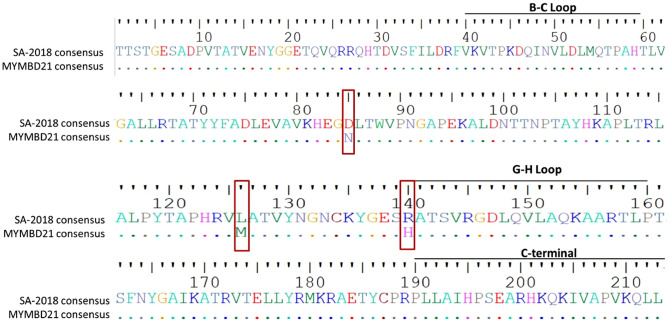
Amino acid variations of MYMBD21 isolate against SA-2018 consensus VP1 sequences. Three amino acid substitutions (D85N, L126M, R140H) are indicated with  shape. VP1 amino acid sequences were aligned and viewed using BioEdit^33^.

Upon mutational analysis, the VP1 protein of MYMBD21 was found to be 99% (210 of 213) identical to that of the SA-2018 as MYMBD21 showed very closeness to this lineage in earlier analyses. Three amino acid substitutions were found (D85N, L126M, R140H) of which one substitution was found at the 140^th^ position of the antigenically critical site, the G-H loop, where arginine (R) was converted to histidine (H) (Fig. 4).

On the other hand, six unique mutations were found in the major and minor antigenic sites of the MYMBD21 isolate compared to previously circulating Bangladeshi isolates, reported in 2013 and 2018. Among them, 2 substitutions were found in the B-C loop (Q45K, A56T) and the G-H loop also contained 2 unique mutations (T141A, N143S). Other mutations were: T96K, and L126M (Supplementary Fig. S5, Supplementary Table S4).

#### Comparative mutational analysis between MYMBD21 and existing vaccine strains

**Figure 5:**
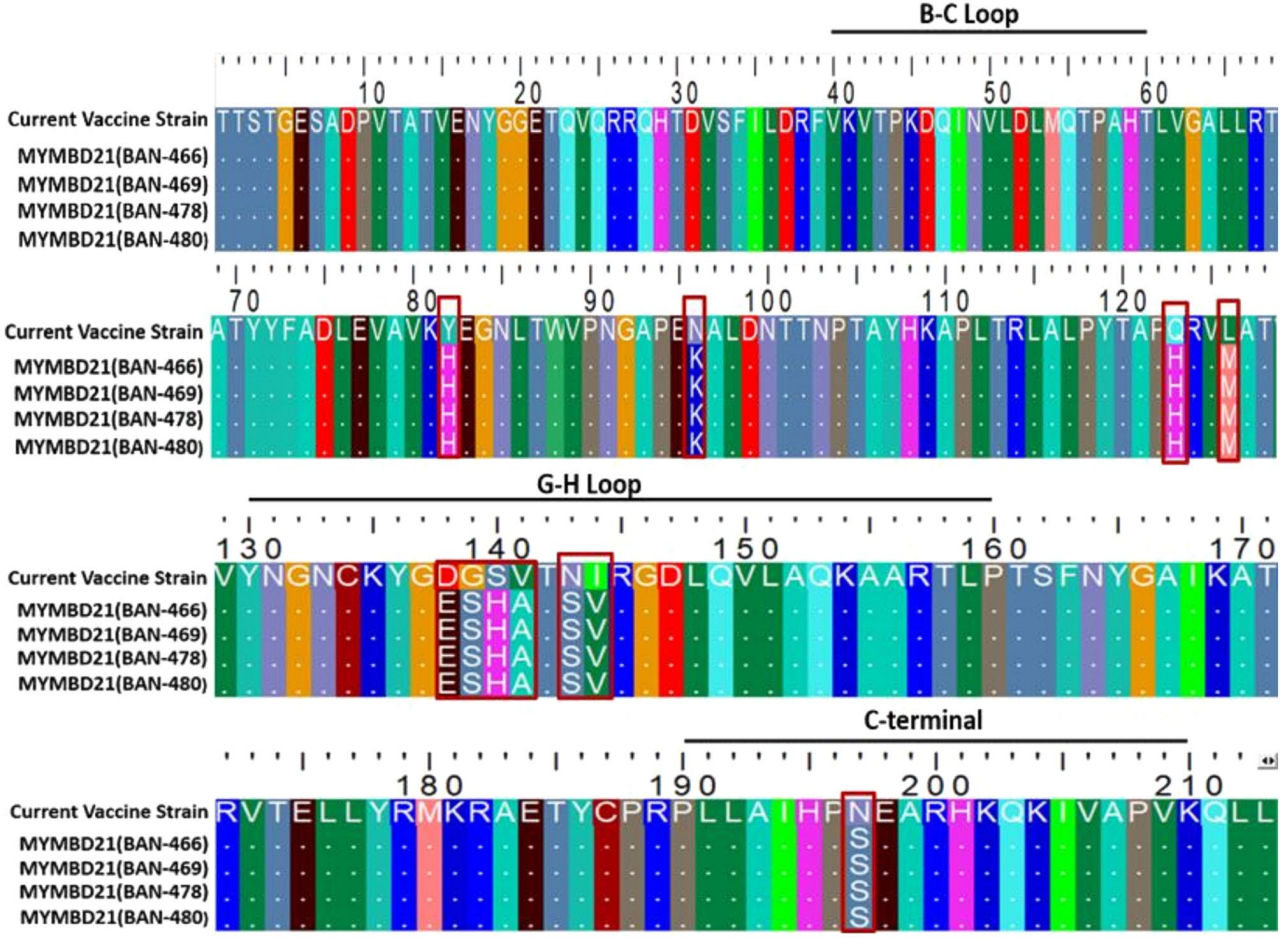
Alignment of the VP1 sequences representing exclusive amino acid substitutions between the current field vaccine strain (O/India/R2/75) and emerging MYMBD21 isolates. Mismatched amino acids are indicated in a shape. The majority of the mutations (6 of 11) occurred in the G-H loop (130–160). VP1 amino acid sequences were aligned and viewed using BioEdit^33^.

**Figure 6:**
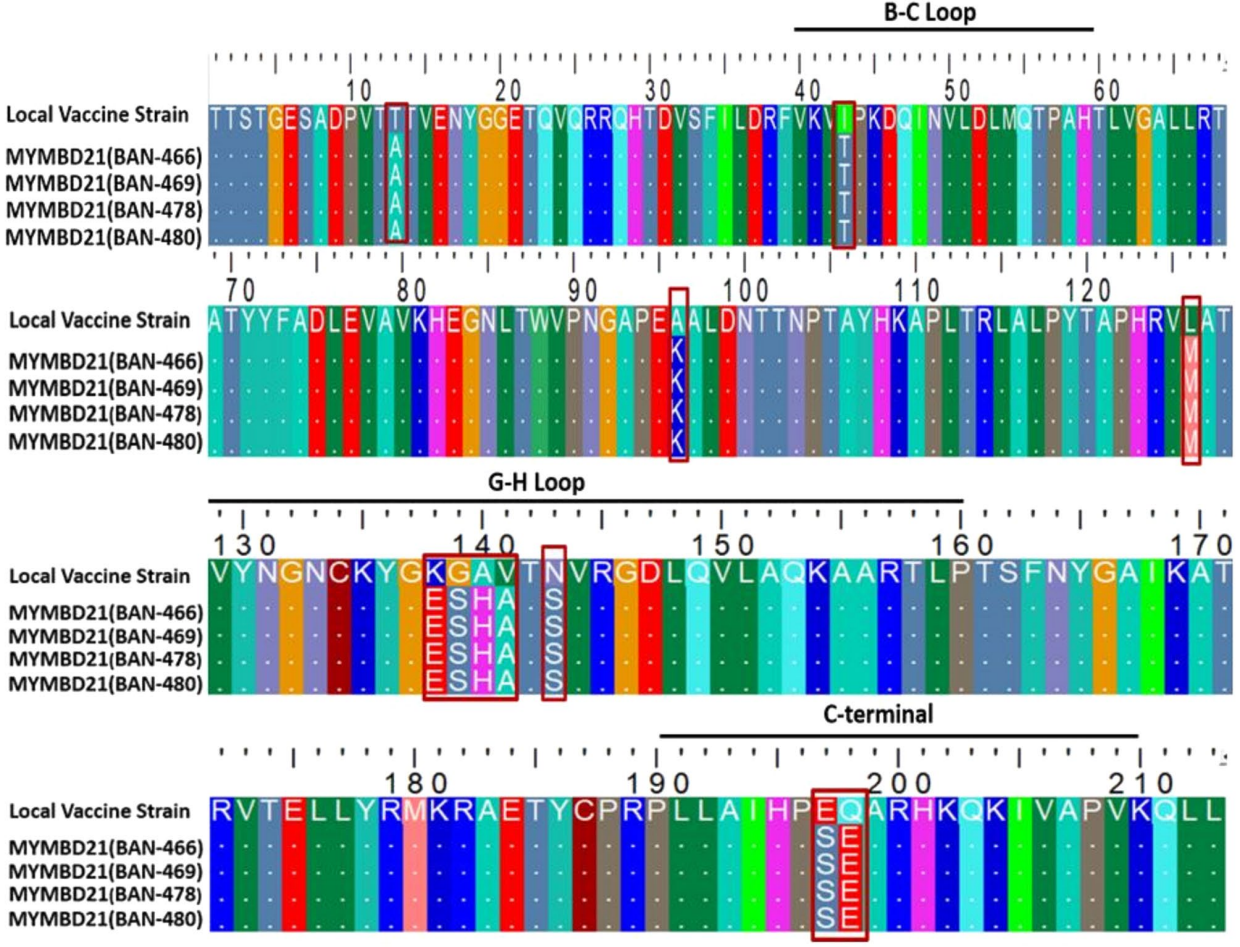
Alignment of the VP1 sequences representing exclusive amino acid substitutions between proposed local vaccine strain (BAN/TA/Dh-301/2016) and emerging MYMBD21 isolates. Mismatched amino acids are indicated with a shape. The majority of the mutations (5 of 11) occurred in the G-H loop (130–160). VP1 amino acid sequences were aligned and viewed using BioEdit^33^ .

The emergence of novel sublineage, MYMBD21 of SA-2018 lineage under serotype O is a significant event as there was no reported cases of FMD by this particular strain in Bangladesh before. VP1 nucleotide sequence of MYMBD21 showed 12–13% divergence from both current field vaccine strain, O/India/R2/75 (accession number: AF204276.1) and proposed local vaccine strain, BAN/TA/Dh-301/2016 (accession number: KY077628.1) (Supplementary Fig. S6, Fig. S7). Comparative mutational analyses were performed to assess the VP1 protein heterogeneity of the MYMBD21 isolates with the currently available vaccine strain, O/India/R2/75 and recently developed local vaccine strain BAN/TA/Dh-301/2016^24^. Between MYMBD21 and O/India/R2/75, 6 out 11 mutations (D138E, G139S, S140H, V141A, N143S, I144V) were found in the G-H loop and a single mutation (N197S) was found in C-terminal site. Between MYMBD21 and BAN/TA/Dh-301/2016, 5 out of 11 mutations (K138E, G139S, A140H, V141A, N143S) were observed in the antigenically critical site, G-H loop and 2 mutations (E197S, Q198E) were found in the C-termini as shown in Figs. 5 and 6, respectively.

### Prediction and evaluation of 3D structure

Three-dimensional (3D) structure of consensus VP1 of MYMBD21 isolate, reference sequences and vaccine strains were built (supplementary Fig. S11, Fig. S13, Fig. S15, Fig. S17, Fig. S19). The Ramachandran plot for all structures showed > 92% of the residues within the favored region. The ProSA analysis of models also showed negative interaction energy and good overall Z-score that are indicative of good quality structures. The result of the quality assessment can be found in supplementary Fig. S10, Fig. S12, Fig. S14, Fig. S16, Fig. S18.

### Conformational change between MYMBD21 and vaccine strains

**Figure 7:**
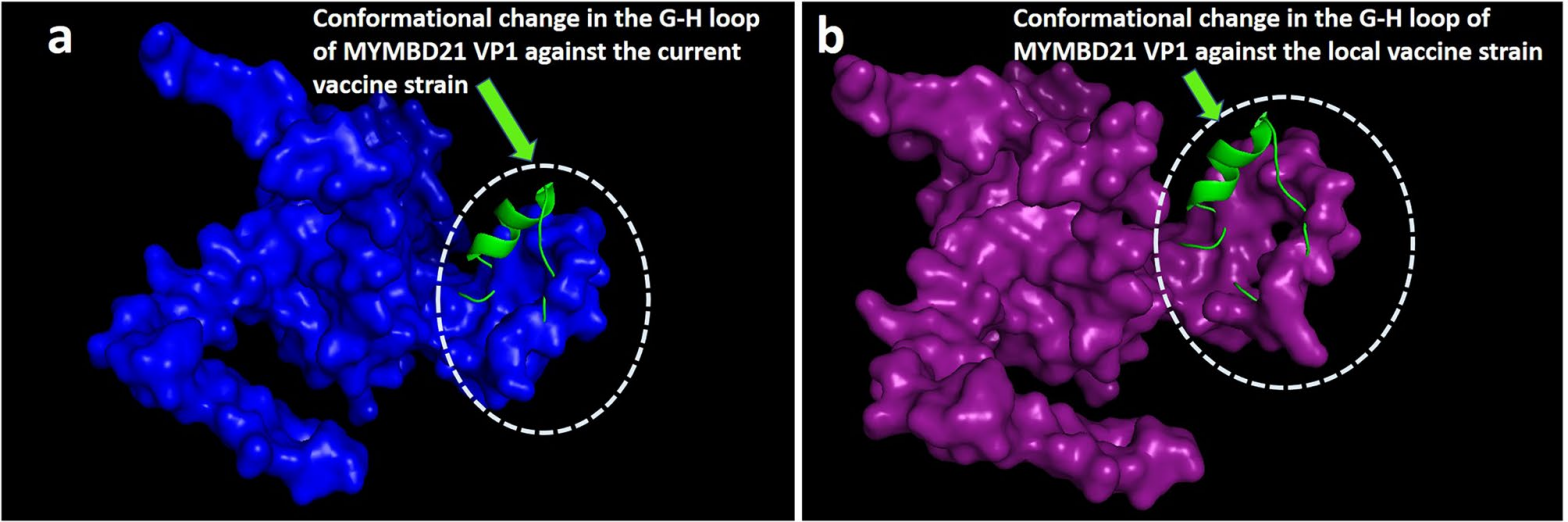
The superimposed 3D structure of VP1 of MYMBD21 and vaccine strains. **(a)** superimposition of VP1 of MYMBD21 and current field vaccine strain (O/India/R2/75) and **(b)** superimposition of VP1 of MYMMBD21 and proposed local vaccine strain (BAN/TA/Dh-301/2016). The protein of MYMBD21 is shown in cartoon style in green color. Both vaccine strains are visualized with surface style but with a different color. The field vaccine strain is colored in blue and the local vaccine strain is shown in deep purple color. The figure shows that the G-H loops of these two strains are displaced from each other and not superimposed. Structures were visualized using PyMOL software^40^.

A major conformational change in the crucial antigenic site, the G-H loop was observed when the VP1 model of MYMBD21 isolate was superimposed on the VP1 model of both currently available field vaccine strain and local vaccine strain. The displacement of the G-H loop in the superimposed model indicates the antigenic heterogeneity of the G-H loop between the novel sublineage, MYMBD21 and both of the existing vaccine strains (Fig. 7).

### Conformational change between MYMBD21 with SA-2018 Indian isolate and uncharacterized Bangladeshi isolate

The superimposition of VP1 structures of MYMBD21 against SA-2018 isolate and uncharacterized Bangladeshi isolate showed some minor changes in the structure due to substitutions in crucial sites but no significant conformational changes in the G-H loop of VP1 occurred. The mutated sites were labelled on the structure. The superimposed structures are given in the Supplementary information file (Fig. S20 a, b).

## Discussion

During recent outbreaks in Bangladesh, FMDV serotypes O and A were circulating with the dominance of serotype O (41 of 46 sequences) over serotype A (5 of 46 sequences), as evidenced by sequencing studies of circulating serotypes based on VP1. Since VP1 sequence-based molecular epidemiology provides important insights into antigenic diversity and virus evolution, this study delved much deeper into the genetic phylogenetic relationships of circulating serotype O strains and comparative mutational analysis against vaccine strains based on the VP1 region of the genome.

VP1-based sequencing study demonstrated the prevalence of Ind-2001 lineage and also the emergence of lineage, SA-2018 in Bangladesh. During the study period, only Ind-2001e (also known as Ind-2001BD1) sublineage was detected under Ind-2001 lineage. The identity of isolates collected in 2019 with the Indian 2018 isolates of Ind-2001BD1 or Ind-2001e sublineage (98–99%) suggests a possible cross boundary transmission of Indian strains into Bangladesh during 2018–19. In the next two years, the changes in sequence identity from 99% to 97% between the circulating strains and Indian isolates was observed which was possibly driven by a selective pressure from both the geographical and demographic factors of this country (Fig. 2; Supplementary Table S1).

In the phylogenetic analysis, the sequences from the Mymensingh district, designated as MYMBD21 in this investigation, revealed an evolutionary link with the 2018 India-reported SA-2018 isolates and prior uncategorized isolates from Bangladesh (BD BAU ML1 2013, BD BAU ML2 2013, BD SI 5 2013, and O/BAN/BLRI/450.2/2018) under the same ME-SA topotype (Fig. 2). However, MYMBD21 developed a completely separate clade from the SA-2018 cluster, differing by 5–6% (Fig. 2; Table 1; Supplementary Fig. S1, Fig. S3) and also from prior uncategorized isolates from Bangladesh with 8% divergence (Fig. S4). The genetic distance of MYMBD21 with SA-2018 (0.062) was lower than that of the Bangladeshi isolates (0.089) and any of the established lineage (Table 1; Supplementary Table S3). The level of identity and genetic distance analyses both suggested that MYMBD21 belongs to the SA-2018 lineage. The genetic distance between previously circulating uncharacterized Bangladeshi isolates and the SA-2018 lineage was 0.101, which was analogous to distances among other established lineages, indicating that these uncategorized Bangladeshi isolates was not under the SA-2018 lineage (Table 1; Supplementary Table S3). This demonstrates that SA-2018 lineage has not previously been discovered in Bangladesh. According to OIE annual country report-2020 (October–December), 13 VP1 sequences of collected samples from India showed the formation of a distinct clade, called 2018 lineage which was later named SA-2018 lineage^19^. In Bangladesh, no study reported the presence of this new lineage in circulation before 2021 until confirmed by this study.

The MYMBD21 isolates, once confirmed to be under the new lineage with the formation of a unique clade, were further analysed for its credibility to be called a new sublineage. BLAST search results and genetic distance analysis showed a considerable divergence (5–6%) from Indian isolates that suggests the possible generation of a new sublineage (Supplementary Fig. S1; Table 1). In multidimensional scaling, MYMBD21 isolates formed a separate cluster from the clusters of other lineages or sublineages, yet were found to be the closest one to SA-2018 cluster (Fig. 3). Formation of a separate evolutionary clade in phylogenetic tree, genetic distance sufficient enough for a separate sublineage combined with distinct cluster formation in MDS plot altogether confirmed that MYMBD21 emerged as a distinct sublineage of SA-2018 lineage in Bangladesh. A large distance from prior Bangladeshi isolates (0.089) further confirms that MYMBD21 is unique to Bangladesh and detected for the first time in this study.

Given the impact of mutation and other associated factors on generation of new sublineages, mutational trend was analysed for MYMBD21 isolates. VP1 amino acid sequence is 99 percent identical (210 of 213) to SA-2018 Indian isolates with 3 observed mutations (D85N, L126M, R140H). Two mutations happened to be prior to the G-H loop at 85^th^ position where negatively charged aspartate (D) was mutated to polar uncharged asparagine (N) and at 126^th^ position where Leucine (L) was converted to Methionine (M) both having hydrophobic side chains. One mutation occurred at the antigenically important G-H loop (R140H) where arginine (R) was transformed to histidine (H) (Fig. 4).

VP1 amino acid sequence of MYMBD21 differed by 5% from both vaccination strains, with 11 alterations (Supplementary Fig. S8, Supplementary Fig. S9; Figs. 5, 6). Between MYMBD21 and the present vaccine strain (O/India/R2/75), 6 out of 11 mutations occurred in the G-H loop including conversion of glycine (G) into serine (S) at position 139, accompanied by replacement of positively charged amino acid with polar amino acid in the G-H loop (Fig. 5). On the other hand, the amino acid variability with the recently reported vaccine strain (BAN/TA/Dh-301/2016) showed mutations in the G-H loop, B-C loop and C-terminal along with others at non-antigenic sites of VP1. 5 out of 11 mutations were found in the G-H loop region (K138E, G139S, A140H, V141A, N143S). Positively charged lysine (K) was mutated into negatively charged glutamate (E) at position 138 and positively charged histidine (H) substituted uncharged alanine (A) at 140th position. G139S showed polarity change. The B-C loop mutation (I43T) changed polarity. In the C-terminal, glutamate was transformed into serine and glutamine into glutamate (E197S and Q198E) (Fig. 6).

Relative comparison of 3D structures of MYMBD21 with vaccine strains (O/India/R2/75; BAN/TA/Dh-301/2016) revealed the G-H loop displacement in the MYMBD21 strain, indicating significant alterations at the antigenic site of VP1 (Fig. 7a, b). Changes in the polarity and charge in the amino acids in the crucial antigenic sites might be attributed to the major conformational changes in G-H loop.

The study shows the first report on the emergence of the SA-2018 lineage in 2021 accompanied by the prevalence of Ind-2001e (or BD1) sublineage during 2019–2021 in Bangladesh. The co-circulation of multiple FMDV serotype O lineages and sublineages, however, can cause comparable cross-immunity between dominant and emergent lineages, which may be crucial for lineage turnover as in endemic areas, susceptible populations develop immunity to the dominant lineage of FMDV rather than the emerging lineage^25^. The extent of a novel lineage's dominance in the future is often difficult to predict, but given the FMDV serotype O lineage's evolutionary history, the SA-2018 lineage can be potential outbreak-initiating lineage in Bangladesh. Additionally, the mutational spectrum of the lineage is evident to reflect a strong indication for the generation of the sublineage O/ME-SA/SA-2018/MYMBD21 in Bangladesh. The impact of the SA-2018 lineage is going to last for a longer time, as the susceptible population will encounter the rapid transmission efficacy of FMDV. It is possible that significant FMD outbreaks might be caused by the novel SA-2018 lineage around the year 2022 given the temporal trend of serotype O lineages emerging and then dominating after a time period of 3–4 years observed in the field. As a result, appropriate measures, such as constant surveillance of FMD outbreaks, appropriate control program and effective vaccination program must be put in place to limit the spread of the disease.

## Conclusions

Intrusion of a new lineage SA-2018 in Bangladesh followed by its emergence as a novel sublineage along with structural heterogeneity with vaccine strains complicates the current FMD situation in Bangladesh. Given the events from the recent past, this sublineage may have the potential to become dominant in near future which requires further genome wide analyses, vaccine candidate selection and stringent control measures to be adopted as soon as possible.

## Methods

### Ethics declarations

Ethical approval for sample collection from cattle and buffalo was taken from the Animal Experimentation Ethical Review Committee (AEERC), Faculty of Biological Sciences, University of Dhaka **(Ref:66/Biol. Sci./2018–19; Date: 14–11-2018)** (Supplementary Fig. S21). Sample collection protocol was in accordance with guidelines and regulations approved by the AEERC and all authors complied with the relevant guidelines. All methods are reported in accordance with ARRIVE guidelines (https://arriveguidelines.org). All the steps of sample handling and processing were performed in a biosafety level 2 laboratory facilities.

### Sample collection

A total of 156 tongue or foot epithelium tissue samples were collected from FMD-suspected cattle and buffalo by a registered veterinary doctor from 2019 to 2021 (Details of the tissue samples are available in Supplementary Table S5). The study area included were: Dhaka, Mymensingh, Tangail, Rajbari, Manikganj, Pabna, Satkhira, Jhenaidah, and Chandpur. Samples were transported from the collection site to the laboratory at 4 °C within 20 h and stored at − 80 °C until processing and testing.

### RNA extraction and synthesis of cDNA

The homogenization of tissue samples and total RNA extraction from tissue were performed in an automated Maxwell 16 system (Promega, USA) using the Maxwell 16 total RNA purification kit (Promega, USA) according to the manufacturer’s instruction. Following the extraction, RNA of the sample was reverse transcribed into complementary DNA (cDNA) using the ImProm-II™ reverse transcription system (Promega, USA).

### Polymerase chain reaction-based amplification of VP1 and VP1 sequencing

VP1-based PCR diagnostic assay was employed for the detection of FMDV positive tissue samples. VP1 region of cDNA was amplified using two sets of primers (VP1UF/NK61, 16F/NK61). The PCR reaction was performed using GoTaq 2 × Hot Start Colorless Master Mix (Promega, USA) with either forward primer VP1UF (5′-GTACTACRCSCAGTAC-3′)^21,22^ or 16F (5′-GAGAACTACGGWGGWGAGAC-3′)^12^ and the reverse primer NK61 (5′-GACATGTCCTCCTGCATCTG-3′)^9^. The PCR products were run on 1.0% agarose gel with a 1 kb-DNA ladder (Promega, USA) for the visualization and detection of FMD positive amplicons. Following detection, the FMDV positive amplified PCR products were purified using the Wizard SV Gel and PCR Clean-Up System (Promega, USA). Then, PCR products were sequenced using BigDye Terminator v3.1 Cycle Sequencing Kit (Applied Biosystems, USA). The VP1 coding sequence quality was analysed in ABI Genetic Analyser (Applied Biosystems, USA). Both forward and reverse sequences were assembled into a single contig using SeqMan version 7.0 (DNASTAR, Inc., Madison, WI, USA). The assembled sequences were compared with other sequences from GenBank^26^ using the basic local alignment search tool, BLAST^27^ to reveal the identity of the isolated virus as well as their serotypes. VP1 sequences of total 46 samples were submitted to the NCBI GenBank database under accession number OP320415-OP320458; OP271696- OP271697 on August, 2022.

### Dataset generation for bioinformatics analysis

A total of 145 representative VP1 reference sequences of FMDV serotype O from Bangladesh and neighbouring countries were included in the dataset for molecular characterization and evolutionary divergence analysis of serotype O isolates of this study (n = 41). Reference sequences for phylogenetic reconstruction were retrieved from the NCBI database. Reference sequences are listed in Supplementary Table S7.

### Phylogeny and Evolutionary divergence analysis

Phylogenetic analysis was performed including 186 VP1 sequences in the final dataset to determine the genotype of the FMDV samples collected in this study during 2019–21 based on the clade formation in MEGA11^28^. The consensus VP1 coding sequences of local FMDV isolates were aligned using the ClustalW program^29^ with the related gene sequences from GenBank. Sequences were accessed from the database on August, 2022. Based on the alignment data, best substitution model Tamura-Nei with discrete Gamma distribution (TN93 + G) was selected based on the lowest BIC (Bayesian Information Criterion) score. Then, phylogenetic Maximum Likelihood tree based on the Tamura-Nei model^30^ were constructed (bootstrap replicates 1000). A discrete Gamma distribution was used to model evolutionary rate differences among sites (5 categories (+ G, parameter = 0.6693)). Alignment gaps, missing data, and ambiguous bases of about fewer than 5% were allowed at any position as all the sequences were not completely aligned on the full range.

The percentage of nucleotide identity between sequences was retrieved from the BLAST^27^ search and was also calculated using a global alignment tool based on the Needleman-Wunsch algorithm^31^ in NCBI.

Genetic divergence analyses of proposed novel sublineage from established lineages or sublineages and previously reported Bangladeshi FMDV isolates were calculated based on the genetic variations to confirm the emergence of novel sublineage or lineage in Bangladesh using the Kimura 2-parameter model^32^ in MEGA11^28^. The rate variation among sites was modelled with a gamma distribution (shape parameter = 1).

### Multidimensional scaling (MDS)

Multidimensional scaling (MDS) was used to visualize the divergence or closeness between the proposed sublineage and other established lineages or sublineages of FMDV. For this analysis, selected reference VP1 sequences from each established lineage or sublineage of serotype O were aligned along with suspected new sublineage and pairwise genetic distance was calculated using the Kimura-2 parameter model^32^ in MEGA11^28^. Using the distance matrix, MDS plotting was performed using IBM SPSS Statistics (Version 26). Each point in the diagram represented one object from the distance matrix of sequences.

### Analysis of VP1 amino acid variations

The VP1 amino acid sequences of samples under study, previously reported reference sequences and vaccine strains were used to analyse mutational spectrum among them. The amino acid was translated based on the standard genetic code after codon-based alignment with MEGA11^28^ and BioEdit^33^ was used for the visualization of variable amino acids and the positions where the substitutions occurred.

### Prediction of the 3-D structure of VP1 Region

Homology modelling of VP1 of a representative FMDV sample, two reference sequences, and two vaccine strains, was performed using the SWISS-MODEL server^34,35^. STML ID 5nem.1.A^36^ was used as a template for modelling the FMDV sample, and STML ID 5ner.1.A^36^ was chosen to model the rest of the structures. The quality of three-dimensional structures was validated using the Ramachandran plot^37^ analysis in *Molprobityv4.4*^38^*.* The quality and the energy criteria of structures were assessed by the ProSA-web^39^ using the PDB files of each model. Using PyMOL^40^, the models were visualized and superimposed for necessary structural analysis.

## Supplementary Information

Supplementary Information.

## Data Availability

Supplementary materials supporting the results of the study are available in this article as Supplementary datasheet and Supplementary information file. VP1 sequences of FMDV isolates included in this study have been submitted to the GenBank databases under accession number OP320415-OP320458; OP271696- OP271697.
