# Supplementary material for: Emergence of a novel sublineage, MYMBD21 under SA-2018 lineage of Foot-and-Mouth Disease Virus serotype O in Bangladesh
Source: Sci Rep. 2023 Jun 17;13:9817. doi: 10.1038/s41598-023-36830-w (PMC10276842; doi:10.1038/s41598-023-36830-w)
Supplement: Supplementary file 1 — Supplementary Information. [file 41598_2023_36830_MOESM1_ESM.pdf]

Supplementary Informations for

Evidence of SA-2018 lineage of Foot-and-Mouth Disease Virus serotype O in Bangladesh with a mutational trend of emerging novel sublineage, MYMBD21

Kazi Alamgir Hossain[1+], Humaira Anjume[1,3+], K.M. Mazharul Alam[1], Ashabul Yeamin[1], Salma Akter[2], M. Anwar Hossain[1,3], Munawar Sultana*[1]

[1]Department of Microbiology, University of Dhaka, Dhaka-1000, Bangladesh.

[2]Present address: Department of Microbiology, Jahangirnagar University, Savar, Dhaka-1342, Bangladesh.

[3]Present address: Jashore University of Science and Technology, Jashore-7408, Bangladesh.

Corresponding Author*: Dr. Munawar Sultana, Professor, Department of Microbiology, University of Dhaka.

E-mail: munawar@du.ac.bd

+Kazi Alamgir Hossain and Humaira Anjume contributed equally

- **Supplementary Tables:**

- **Evolutionary divergence analysis**

Genotyping of Ind-2001 lineage:

[The identities between two groups were determined by BLAST search and using a global alignment tool based on the Needleman-Wunsch algorithm in NCBI]

Supplementary Table S1 Nucleotide Identity and Evolutionary Divergence between Groups

Sub-lineage/isolate	Sub-lineage	Identity from BLAST search (%)	Genetic distance calculated in MEGA11	Standard error
Ind-2001BD1	Ind-2001e (Indian isolates)	97%	0.026	0.005
2019 samples (This study)	Ind-2001e (Indian isolates)	98-99%	0.018	0.003
2019 samples (This study)	Ind-2001BD1 (Bangladeshi isolates)	97%	0.032	0.006
2020-21 samples (This study)	Ind-2001e (Indian isolates)	97%	0.046	0.008
2020-21 samples (This study)	Ind-2001BD1 (Bangladeshi isolates)	97%	0.035	0.007

The genetic distance calculated between Ind-2001BD1 and Ind-2001e in MEGA11 using the Kimura-2 parameter model was 0.026 which was lower than the lowest distance (0.044) among established sub-lineages which confirms that Ind-2001BD1 and Ind-2001e isolates are not two distinct sub-lineages of the Ind-2001 lineage rather the same sub-lineage **(Table S2).**

Supplementary Table S2 Evolutionary Divergence between established sub-lineages of Ind-2001 lineage and groups of isolates calculated in MEGA11[1].

	1	2	3	4	5	6	7	8	9
1. 2020-21 isolates		0.010	0.007	0.013	0.016	0.015	0.017	0.014	0.009
2. 2019 isolates	0.058		0.006	0.014	0.017	0.015	0.018	0.013	0.003
3. Ind-2001BD1	0.035	0.032		0.012	0.015	0.013	0.016	0.012	0.005
4. Ind-2001BD2	0.084	0.090	0.079		0.013	0.011	0.014	0.011	0.013
5. Ind-2001a	0.114	0.118	0.103	0.079		0.008	0.011	0.012	0.015
6. Ind-2001b	0.105	0.103	0.089	0.068	0.044		0.012	0.013	0.013
7. Ind-2001c	0.119	0.124	0.115	0.095	0.069	0.078		0.014	0.016
8. Ind-2001d	0.099	0.091	0.085	0.073	0.088	0.092	0.106		0.011
9. Ind-2001e	0.046	0.018	0.026	0.084	0.105	0.093	0.114	0.082	

Supplementary Table S3 Evolutionary Divergence among groups of established lineages (PanAsia, PanAsia-2, SA-2018) of serotype O, MYMBD21 and uncharacterized Bangladeshi isolates calculated in MEGA11[1]:

	1	2	3	4	5
1. PanAsia		0.009	0.013	0.015	0.014
2. PanAsia-2	0.102		0.013	0.014	0.012
3. Uncharacterized BDisolates	0.110	0.119		0.014	0.013
4. SA-2018	0.129	0.124	0.101		0.010
5. MYMBD21	0.108	0.105	0.089	0.062	

❖ **Amino acid variations**

Supplementary Table S4. Amino acid substitutions in the VP1 region of novel sublineage MYMBD21 isolates against previously reported Bangladeshi isolates (BD_BAU_ML1_2013; BD_BAU_ML2_2013; O/BAN/BLRI/450.2/2018; BD_SI _5_2013) and SA-2018.

Region in VP1	Antigenic sites in the region	Position	BD_BAU_ML1_2013; BD_BAU_ML2_2013; BD_SI _5_2013	O/BAN/BLRI /450.2/2018	MYMBD21 (2021)	SA-2018 (Reported in India)
B-C loop (43-59) [2]	43-48 [3]	45	Q	Q	K	K
		56	A	A	T	T
		57	P	L	P	P
		69	G, A	A	A	A
		83	E, K	K	E	E
		85	N	N	N	D
		90	P	R	P	P
		96	T	T	K	K
		126	L	L	M	L
G-H loop (130-160) [4]	138-154 [5]	140	H	H	H	R
		141	T	T	A	A
		143	N	N	S	S
C-terminal (190-213) [6]	194-213 [6]	197	S	G	S	S
		210	K, N	N	K	K

Supplementary Table S5 Detailed information of samples included in this study and the result of VP1 based PCR of the sample for detection of the presence of FMDV

Sample ID	Date of Collection	District	Host	Age (Years)	Sex	Breed	Farming system	Sample source (Feet/Tongue)	FMDV detection by VP1 based PCR
BAN/DH/Dh-326/2019	8/4/2019	Dhaka	Cattle	2	Male	Cross	Semi-intensive	Tongue Tissue	Negative
BAN/DH/Dh-327/2019	8/4/2019	Dhaka	Cattle	2.5	Male	Cross	Semi-intensive	Tongue Tissue	Positive
BAN/DH/Dh-328/2019	8/6/2019	Dhaka	Cattle	3	Male	Cross	Intensive	Tongue Tissue	Negative
BAN/RAJ/Pa-329/2019	9/3/2019	Rajbari	Cattle	1.5	Male	Cross	Intensive	Tongue Tissue	Positive
BAN/RAJ/Pa-330/2019	9/3/2019	Rajbari	Cattle	1.5	Female	Cross	Intensive	Tongue Tissue	Positive
BAN/RAJ/Pa-331/2019	9/3/2019	Rajbari	Cattle	1.5	Male	Cross	Semi-intensive	Foot Tissue	Positive
BAN/RAJ/Pa-332/2019	9/3/2019	Rajbari	Cattle	4	Female	Cross	Semi-intensive	Tongue Tissue	Positive
BAN/RAJ/Pan-333/2019	9/4/2019	Rajbari	Cattle	4.5	Female	Cross	Semi-intensive	Tongue Tissue	Positive
BAN/RAJ/Kal-334/2019	9/4/2019	Rajbari	Cattle	1.5	Female	Cross	Semi-intensive	Tongue Tissue	Positive
BAN/RAJ/Pa-335/2019	9/4/2019	Rajbari	Cattle	2	Male	Cross	Semi-intensive	Tongue Tissue	Positive
BAN/RAJ/Pa-336/2019	9/4/2019	Rajbari	Goat	3	Female	Local	Semi-intensive	Foot Tissue	Positive
BAN/RAJ/Pa-337/2019	9/4/2019	Rajbari	Cattle	2	Female	Cross	Intensive	Foot Tissue	Positive
BAN/RAJ/Kal-338/2019	9/5/2019	Rajbari	Cattle	0.5	Male	Cross	Semi-intensive	Tongue Tissue	Negative
BAN/RAJ/Ka-339/2019	9/5/2019	Rajbari	Cattle	3	Female	Local	Semi-intensive	Tongue Tissue	Negative
BAN/MA/Do-340/2019	9/11/2019	Manikgonj	Goyal	1	Female	Local	Intensive	Tongue Tissue	Positive
BAN/MA/Sh-341/2019	9/12/2019	Manikgonj	Cattle	1.5	Male	Cross	Semi-intensive	Tongue Tissue	Positive
BAN/MA/Sa-342/2019	9/12/2019	Manikgonj	Cattle	2	Male	Cross	Semi-intensive	Tongue Tissue	Positive

BAN/MA/Sa-343/2019	9/12/2019	Manikgonj	Cattle	2	Male	Cross	Semi-intensive	Tongue Tissue	Positive
BAN/MA/Sh-344/2019	14/9/2019	Manikgonj	Cattle	6	Female	Local	Semi-intensive	Tongue Tissue	Positive
BAN/MA/Sh-345/2019	14/9/2019	Manikgonj	Cattle	2	Female	Local	Semi-intensive	Tongue Tissue	Positive
BAN/MA/Sh-346/2019	14/9/2019	Manikgonj	Cattle	2 months	Female	Local	Semi-intensive	Foot Tissue	Negative
BAN/PA/At-347/2019	17/9/2019	Pabna	Cattle	5	Female	Local	Semi-intensive	Tongue Tissue	Positive
BAN/PA/At-348/2019	17/9/2019	Pabna	Buffalo	2	Male	Local	Semi-intensive	Tongue Tissue	Positive
BAN/PA/At-349/2019	17/9/2019	Pabna	Cattle	1.5	Male	Cross	Semi-intensive	Foot Tissue	Positive
BAN/PA/At-350/2019	17/9/2019	Pabna	Cattle	6	Female	Cross	Semi-intensive	Tongue Tissue	Positive
BAN/PA/At-351/2019	17/9/2019	Pabna	Cattle	7	Female	Cross	Intensive	Tongue Tissue+ Foot Tissue	Positive
BAN/PA/At-352/2019	18/9/2019	Pabna	Cattle	5	Female	Cross	Semi-intensive	Tongue Tissue	Positive
BAN/PA/At-353/2019	18/9/2019	Pabna	Cattle	1.5	Male	Cross	Semi-intensive	Tongue Tissue	Negative
BAN/PA/At-354/2019	18/9/2019	Pabna	Cattle	1.5	Male	Local	Semi-intensive	Foot Tissue	Negative
BAN/PA/At-355/2019	18/9/2019	Pabna	Cattle	5	Female	Cross	Semi-intensive	Tongue Tissue	Positive
BAN/PA/at-356/2019	18/9/2019	Pabna	Cattle	6	Female	Cross	Semi-intensive	Foot Tissue	Positive
BAN/RA/Ka-357/2019	10/2/2019	Rajbari	Cattle	4.5	Female	Cross	Semi-intensive	Tongue Tissue	Positive
BAN/RA/Ka-358/2019	10/3/2019	Rajbari	Cattle	6 months	Male	Local	Semi-intensive	Tongue Tissue	Positive
BAN/DH/Dh-359/2019	15/10/2019	Dhaka	Cattle	3	Male	Cross	Semi-intensive	Tongue Tissue+Foot Tissue	Positive
BAN/DH/Dh-360/2019	15/10/2019	Dhaka	Cattle	2.5	Male	Cross	Semi-intensive	Tongue Tissue	Positive

BAN/DH/Dh-361/2019	15/10/2019	Dhaka	Cattle	1.5	Male	Local	Semi-intensive	Tongue Tissue	Positive
BAN/DH/Dh-362/2019	15/10/2019	Dhaka	Cattle	1.5	Male	Cross	Semi-intensive	Tongue Tissue	Positive
BAN/DH/Dh-363/2019	19/10/2019	Dhaka	Cattle	2.5	Female	Cross	Intensive	Tongue Tissue	Positive
BAN/DH/Dh-364/2019	19/10/2019	Dhaka	Cattle	5	Female	Cross	Intensive	Tongue Tissue	Positive
BAN/MA/Ma-365/2019	19/10/2019	Manikgonj	Cattle	6	Female	Local	Semi-intensive	Tongue Tissue	Positive
BAN/MA/Ma-366/2019	19/10/2019	Manikgonj	Cattle	2	Female	Cross	Semi-intensive	Tongue Tissue	Positive
BAN/DH/Dh-367/2019	23/10/2019	Dhaka	Cattle	2.5	Male	Cross	Intensive	Tongue Tissue	Positive
BAN/MA/Si-368/2019	23/10/2019	Manikgonj	Cattle	1.5	Male	Cross	Semi-intensive	Tongue Tissue	Positive
BAN/MA/SI-369/2019	23/10/2019	Manikgonj	Cattle	1.5	Male	Cross	Semi-intensive	Foot Tissue	Positive
BAN/MA/Ma-370/2019	26/10/2019	Manikgonj	Cattle	1	Male	Cross	Semi-intensive	Tongue Tissue	Negative
BAN/DH/Dh-371/2019	26/10/2019	Dhaka	Cattle	2.5	Male	Cross	Intensive	Tongue Tissue	Positive
BAN/DH/Dh-372/2019	26/10/2019	Dhaka	Cattle	2.5	Male	Cross	Intensive	Tongue Tissue	Negative
BAN/DH/Dh-373/2020	14/01/2020	Dhaka	Cattle	3	Male	Cross	Intensive	Tongue Tissue	Negative
BAN/DH/Dh-374/2020	14/01/2020	Dhaka	Cattle	6	Female	Cross	Intensive	Tongue Tissue	Positive
BAN/DH/Dh-375/2020	14/01/2020	Dhaka	Cattle	5	Female	Cross	Intensive	Tongue Tissue	Positive
BAN/DH/Dh-376/2020	14/01/2020	Dhaka	Cattle	1.5	Male	Cross	Intensive	Tongue Tissue	Negative
BAN/DH/Dh-377/2020	14/01/2020	Dhaka	Cattle	1	Male	Cross	Intensive	Tongue Tissue	Positive
BAN/CH/Ch-378/2020	3/10/2020	Chandpur	Cattle	8	Female	Cross	Intensive	Tongue + Foot Tissue	Positive
BAN/CH/Ch-379/2020	3/10/2020	Chandpur	Cattle	6 months	Female	Cross	Intensive	Tongue Tissue	Negative
BAN/CH/Ch-380/2020	3/10/2020	Chandpur	Cattle	6 months	Male	Cross	Intensive	Tongue Tissue	Negative
BAN/CH/Ch-381/2020	3/10/2020	Chandpur	Cattle	6	Female	Cross	Intensive	Tongue Tissue	Positive
BAN/CH/Ch-382/2020	3/10/2020	Chandpur	Cattle	10 months	Female	Cross	Intensive	Tongue Tissue	Negative

BAN/CH/Ch-383/2020	3/10/2020	Chandpur	Cattle	6	Female	Cross	Intensive	Tongue Tissue	Positive
BAN/CH/Ch-384/2020	3/10/2020	Chandpur	Cattle	8 months	Male	Cross	Intensive	Tongue Tissue	Negative
BAN/CH/Ch-385/2020	3/10/2020	Chandpur	Cattle	6 months	Female	Cross	Intensive	Tongue Tissue	Negative
BAN/CH/Ch-386/2020	3/10/2020	Chandpur	Cattle	2.5	Female	Cross	Intensive	Tongue Tissue	Positive
BAN/CH/Ch-387/2020	3/10/2020	Chandpur	Cattle	5	Female	Cross	Intensive	Foot Tissue	Positive
BAN/DH/Ke-388/2020	15/11/2020	Dhaka	Cattle	4	Female	Cross	Intensive	Tongue Tissue	Positive
BAN/DH/Ke-389/2020	15/11/2020	Dhaka	Cattle	6 months	Female	Cross	Intensive	Tongue Tissue	Positive
BAN/DH/Ke-390/2020	15/11/2020	Dhaka	Cattle	1.5	Female	Cross	Intensive	Tongue Tissue	Positive
BAN/DH/Ke-391/2020	15/11/2020	Dhaka	Cattle	2	Male	Cross	Intensive	Tongue Tissue	Positive
BAN/DH/Ke-392/2020	15/11/2020	Dhaka	Cattle	7	Female	Cross	Intensive	Tongue Tissue	Positive
BAN/DH/Ke-393/2020	15/11/2020	Dhaka	Cattle	1.5	Male	Cross	Intensive	Tongue Tissue	Positive
BAN/DH/Ke-394/2020	15/11/2020	Dhaka	Cattle	1	Male	Cross	Intensive	Tongue Tissue	Positive
BAN/DH/Ke-395/2020	15/11/2020	Dhaka	Cattle	7	Female	Cross	Intensive	Tongue Tissue	Positive
BAN/TA/Mi-396/2020	22/11/2020	Tangail	Cattle	2.5	Male	Cross	Intensive	Foot Tissue	Positive
BAN/TA/Mi-397/2020	22/11/2020	Tangail	Cattle	8	Female	Cross	Semi-intensive	Tongue Tissue	Positive
BAN/TA/Mi-398/2020	22/11/2020	Tangail	Cattle	6	Female	Cross	Semi-intensive	Tongue Tissue	Positive
BAN/TA/Mi-399/2020	22/11/2020	Tangail	Cattle	2.5	Male	Cross	Semi-intensive	Tongue Tissue	Negative
BAN/DH/Sa-400/2020	25/11/2020	Dhaka	Cattle	2	Female	Cross	Intensive	Tongue Tissue	Positive
BAN/DH/Sa-401/2020	25/11/2020	Dhaka	Cattle	2	Male	Cross	Intensive	Tongue Tissue	Positive
BAN/MA/Sh-402/2021	1/3/2021	Manikgonj	Cattle	4 months	Female	Cross	Intensive	Tongue Tissue	Positive
BAN/MA/Sh-403/2021	1/3/2021	Manikgonj	Cattle	3 months	Female	Cross	Intensive	Tongue Tissue	Positive
BAN/MA/Sh-404/2021	1/3/2021	Manikgonj	Cattle	3	Male	Cross	Intensive	Tongue Tissue	Negative
BAN/DH/Dh-405/2021	1/3/2021	Dhaka	Cattle	6	Female	Cross	Intensive	Tongue Tissue	positive
BAN/DH/Dh-406/2021	1/3/2021	Dhaka	Cattle	2	Male	Cross	Intensive	Tongue Tissue	Positive

BAN/DH/Dh-407/2021	2/1/2021	Dhaka	Cattle	3	Female	Cross	Semi-intensive	Tongue Tissue	Negative
BAN/ DH/Dh-408/2021	2/1/2021	Dhaka	Cattle	6	Female	Cross	Semi-intensive	Tongue Tissue	Negative
BAN/DH/Dh-409/2021	2/1/2021	Dhaka	Cattle	2	Male	Cross	Semi-intensive	Tongue Tissue	N/A
BAN/DH/Dh-410/2021	2/1/2021	Dhaka	cattle	2	Male	cross	Intensive	Tongue Tissue	Positive
BAN/DH/Dh-411/2021	2/1/2021	Dhaka	Cattle	1.5	Male	Cross	Intensive	Tongue Tissue	Negative
BAN/DH/Dh-412/2021	2/2/2021	Dhaka	Cattle	1.5	Female	Cross	Semi-intensive	Tongue Tissue	Negative
BAN/DH/Dh-413/2021	2/2/2021	Dhaka	Cattle	1	Female	Cross	Semi-intensive	Tongue Tissue	Negative
BAN/DH/Dh-414/2021	2/2/2021	Dhaka	Cattle	2	Male	Cross	Semi-intensive	Tongue Tissue	Negative
BAN/DH/Dh-415/2021	2/2/2021	Dhaka	Cattle	2.5	Male	Cross	Semi-intensive	Tongue Tissue	Negative
BAN/DH/Dh-416/2021	2/8/2021	Dhaka	Cattle	5	Female	Cross	Intensive	Tongue Tissue	Positive
BAN/DH/Dh-417/2021	2/8/2021	Dhaka	Cattle	10	Female	Cross	Intensive	Tongue Tissue	Positive
BAN/DH/Dh-418/2021	2/8/2021	Dhaka	Cattle	2	Male	Cross	Intensive	Tongue Tissue	Positive
BAN/MA/Ma-419/2021	2/12/2021	Manikgonj	Cattle	1	Male	Cross	Semi-intensive	Tongue Tissue	Negative
BAN/MA/Ma-420/2021	2/12/2021	Manikgonj	Cattle	6	Female	Cross	Semi-intensive	Tongue Tissue	N/A
BAN/SA/Sa-421/2021	3/3/2021	Satkhira	Cattle	4	Female	Cross	Intensive	Tongue Tissue	N/A
BAN/SA/Sa-422/2021	3/3/2021	Satkhira	Cattle	1.5	Female	Cross	Intensive	Tongue Tissue	Negative
BAN/SA/Sa-423/2021	3/3/2021	Satkhira	Cattle	2	Male	Cross	Semi-intensive	Tongue Tissue	Positive
BAN/SA/Sa-424/2021	3/3/2021	Satkhira	Cattle	2	Male	Cross	Semi-intensive	Tongue Tissue	Negative
BAN/SA/Sa-425/2021	3/3/2021	Satkhira	Cattle	6	Female	Cross	Intensive	Tongue Tissue	Positive
BAN/SA/Sa-426/2021	3/3/2021	Satkhira	Cattle	5	Female	Cross	Intensive	Tongue Tissue	Positive
BAN/SA/Sa-427/2021	3/4/2021	Satkhira	Cattle	2	Male	Cross	Intensive	Tongue Tissue	Negative
BAN/SA/Sa-428/2021	3/4/2021	Satkhira	Cattle	2.5	Male	Cross	Intensive	Tongue Tissue	N/A

BAN/SA/Sa-429/2021	3/4/2021	Satkhira	Cattle	4	Female	Cross	Intensive	Tongue Tissue	Negative
BAN/SA/Sa-430/2021	3/4/2021	Satkhira	Cattle	4	Female	Cross	Semi-intensive	Tongue Tissue	Positive
BAN/SA/Sa-431/2021	3/4/2021	Satkhira	Cattle	6	Female	Local	Semi-intensive	Tongue Tissue	Positive
BAN/SA/Sa-432/2021	3/4/2021	Satkhira	Cattle	5	Female	Cross	Semi-intensive	Tongue Tissue	Positive
BAN/SA/Sa-433/2021	3/4/2021	Satkhira	Cattle	2	Male	Local	Semi-intensive	Tongue Tissue	Positive
BAN/SA/Sa-434/2021	3/4/2021	Satkhira	Cattle	2	Male	Local	Semi-intensive	Tongue Tissue	Negative
BAN/SA/Sa-435/2021	3/4/2021	Satkhira	Cattle	2	Female	Cross	Intensive	Tongue Tissue	Positive
BAN/SA/Sa-436/2021	3/4/2021	Satkhira	Cattle	2.5	Male	Cross	Intensive	Tongue Tissue	Negative
BAN/SA/Sa-437/2021	3/4/2021	Satkhira	Cattle	2	ma	Local	Intensive	Tongue Tissue	N/A
BAN/SA/Sa-438/2021	3/4/2021	Satkhira	Cattle	4	Female	Local	Semi-intensive	Tongue Tissue	N/A
BAN/SA/Sa-439/2021	3/4/2021	Satkhira	Cattle	1	Female	Cross	Semi-intensive	Tongue Tissue	Negative
BAN/SA/Sa-440/2021	3/4/2021	Satkhira	Cattle	2	ma	Cross	Intensive	Tongue Tissue	Positive
BAN/SA/Sa-441/2021	3/4/2021	Satkhira	Cattle	2.5	Male	Local	Intensive	Tongue Tissue	Negative
BAN/SA/Sa-442/2021	3/4/2021	Satkhira	Cattle	2	Female	Cross	Intensive	Tongue Tissue	N/A
BAN/SA/Sa-443/2021	3/4/2021	Satkhira	Cattle	1.5	Female	Cross	Intensive	Tongue Tissue	N/A
BAN/SA/Sa-444/2021	3/4/2021	Satkhira	Cattle	5	Female	Cross	Intensive	Tongue Tissue	N/A
BAN/SA/Sa-445/2021	3/4/2021	Satkhira	Cattle	7	Female	Cross	Intensive	Tongue Tissue	Negative
BAN/SA/Sa-446/20021	3/4/2021	Satkhira	Cattle	1.5	Male	Cross	Intensive	Tongue Tissue	Positive
BAN/SA/Sa-447/2021	3/4/2021	Satkhira	Cattle	2.5	male	Cross	Intensive	Tongue Tissue	Positive
BAN/SA/Sa-448/2021	3/4/2021	Satkhira	Cattle	4	Female	Cross	Intensive	Tongue Tissue	Negative
BAN/SA/Sa-449/2021	3/4/2021	Satkhira	Cattle	2	Male	Cross	Intensive	Tongue Tissue	Negative
BAN/JH/Jh-450/2021	20/03/2021	Jhinaidah	Cattle	3	Male	Cross	Intensive	Tongue Tissue	Negative
BAN/JH/Jh-451/2021	20/03/2021	Jhinaidah	Cattle	2	Female	Cross	Semi-intensive	Tongue Tissue	Negative

BAN/JH/Jh-452/2021	20/03/2021	Jhinaidah	Cattle	3	Female	Cross	Semi-intensive	Tongue Tissue	Positive
BAN/JH/Jh-453/2021	20/03/2021	Jhinaidah	Cattle	4	Female	Cross	Semi-intensive	Tongue Tissue	Positive
BAN/JH/Jh-454/2021	20/03/2021	Jhinaidah	Cattle	2	Female	Cross	Semi-intensive	Foot Tissue	Positive
BAN/JH/Jh-455/2021	20/03/2021	Jhinaidah	Cattle	3	Female	Cross	Semi-intensive	Tongue Tissue	Positive
BAN/JH/Jh-456/2021	20/03/2021	Jhinaidah	Cattle	3	Female	Cross	Semi-intensive	Foot Tissue	Positive
BAN/JH/Jh-457/2021	20/03/2021	Jhinaidah	Cattle	1	Female	Cross	Semi-intensive	Tongue Tissue	Positive
BAN/JH/Jh-458/2021	20/03/2021	Jhinaidah	Cattle	1	Female	Cross	Semi-intensive	Foot Tissue	Positive
BAN/JH/Jh-459/2021	20/03/2021	Jhinaidah	Cattle	2.5	Male	Cross	Semi-intensive	Tongue Tissue	Positive
BAN/JH/Jh-460/2021	21/03/2021	Jhinaidah	Cattle	3	Female	Cross	Semi-intensive	Tongue Tissue	Negative
BAN/JH/Ka-461/2021	21/03/2021	Jhinaidah	Cattle	6	Female	Local	Semi-intensive	Tongue Tissue	Positive
BAN/JH/Ka-462/2021	21/03/2021	Jhinaidah	Cattle	2	Male	Cross	Semi-intensive	Tongue Tissue	Positive
BAN/JH/Ka-463/2021	21/03/2021	Jhinaidah	Cattle	4	Female	Local	Semi-intensive	Tongue Tissue	Positive
BAN/JH/Ka-464/2021	21/03/2021	Jhinaidah	Cattle	2.5	Female	Local	Semi-intensive	Tongue Tissue	Positive
BAN/JH/Ka-465/2021	21/03/2021	Jhinaidah	Cattle	4	Female	Cross	Semi-intensive	Tongue Tissue	Negative
BAN/MY/My466/2021	30/12/2021	Mymensingh	Cattle	3	Male	Local	Intensive	Tongue Tissue	Positive
BAN/MY/My467/2021	30/12/2021	Mymensingh	Cattle	4	Male	Local	Intensive	Tongue Tissue	Positive
BAN/MY/My/468/2021	30/12/2021	Mymensingh	Cattle	6	Female	Cross	Intensive	Tongue Tissue	Negative
BAN/MY/My/469/2021	30/12/2021	Mymensingh	Cattle	5	Female	Cross	Intensive	Tongue Tissue	Positive
BAN/MY/My/470/2021	30/12/2021	Mymensingh	Cattle	1	Female	Cross	Intensive	Tongue Tissue	Positive
BAN/MY/My/471/2021	30/12/2021	Mymensingh	Cattle	2	Female	Cross	Intensive	Tongue Tissue	Positive
BAN/MY/My/472/2021	30/12/2021	Mymensingh	Cattle	6	Female	Cross	Intensive	Tongue Tissue	Positive

BAN/MY/My/473/2021	30/12/2021	Mymensingh	Cattle	2.5	Female	Cross	Intensive	Foot Tissue	Positive
BAN/MY/My/474/2021	30/12/2021	Mymensingh	Cattle	5	Female	Cross	Intensive	Tongue Tissue	Positive
BAN/MY/My/475/2021	30/12/2021	Mymensingh	Cattle	3	Female	Cross	Intensive	Tongue Tissue	Positive
BAN/MY/My/476/2021	30/12/2021	Mymensingh	Cattle	5	Female	Cross	Intensive	Tongue Tissue	Positive
BAN/MY/My/477/2021	30/12/2021	Mymensingh	Cattle	4	Female	Cross	Intensive	Tongue Tissue	Positive
BAN/MY/My/478/2021	30/12/2021	Mymensingh	Cattle	1	Female	Cross	Intensive	Tongue Tissue	Positive
BAN/MY/My/479/2021	30/12/2021	Mymensingh	Cattle	1.5	Female	Cross	Intensive	Tongue Tissue	Positive
BAN/MY/My/480/2021	30/12/2021	Mymensingh	Cattle	1.5	Male	Local	Semi-intensive	Foot Tissue	Positive
BAN/MY/My/481/2021	30/12/2021	Mymensingh	Cattle	4	Female	Local	Semi-intensive	Tongue Tissue	Positive

Spplementary Table S6 Accession number of VP1 sequences of the sample included in this study

Sample ID	GenBank Accession No.	Serotype
BAN/RAJ/Pa-329/2019	OP320415	O
BAN/RAJ/Pa-332/2019	OP320416	O
BAN/RAJ/Ka-333/2019	OP320417	O
BAN/RAJ/Pa-335/2019	OP320418	O
BAN/MA/Sh-341/2019	OP320419	O
BAN/MA/Sa-343/2019	OP320420	O
BAN/PA/At-347/2019	OP320421	O
BAN/PA/At-348/2019	OP320422	O
BAN/PA/At-350/2019	OP320423	O
BAN/PA/At-352/2019	OP320424	O
BAN/PA/At-355/2019	OP320425	O
BAN/RAJ/Ka-357/2019	OP320426	O

BAN/DH/Dh-359/2019	OP320427	O
BAN/DH/Dh-360/2019	OP320428	A
BAN/MA/Ma-362/2019	OP320429	A
BAN/DH/Dh-363/2019	OP320430	O
BAN/MA/Ma-365/2019	OP320431	O
BAN/MA/Ma-366/2019	OP320432	O
BAN/MA/Si-369/2019	OP320433	O
BAN/DH/Dh-377/2020	OP320434	O
BAN/CH/Ch-381/2020	OP320435	A
BAN/CH/Ch-386/2020	OP320436	A
BAN/DH/Ke-391/2020	OP320437	A
BAN/DH/Ke-393/2020	OP320438	O
BAN/DH/Ke-395/2020	OP320439	O
BAN/TA/Mi-396/2020	OP271696	O
BAN/DH/Sa-400/2020	OP320440	O
BAN/DH/Dh-410/2021	OP271697	O
BAN/DH/Dh-416/2021	OP320441	O
BAN/DH/Dh-417/2021	OP320442	O
BAN/DH/Dh-418/2021	OP320443	O
BAN/SA/Sa-423/2021	OP320444	O
BAN/SA/Sa-426/2021	OP320445	O
BAN/SA/Sa-431/2021	OP320446	O
BAN/SA/Sa-432/2021	OP320447	O
BAN/SA/Sa-433/2021	OP320448	O

**BAN/SA/Sa-440/2021**	**OP320449**	**O**
**BAN/JH/Jh-454/2021**	**OP320450**	**O**
**BAN/JH/Jh-455/2021**	**OP320451**	**O**
**BAN/JH/Jh-457/2021**	**OP320452**	**O**
**BAN/JH/Ka-461/2021**	**OP320453**	**O**
**BAN/JH/Ka-464/2021**	**OP320454**	**O**
**BAN/MY/My-466/2021**	**OP320455**	**O**
**BAN/MY/My-469/2021**	**OP320456**	**O**
**BAN/MY/My-478/2021**	**OP320457**	**O**
**BAN/MY/My-480/2021**	**OP320458**	**O**

Supplementary Table S7 List of reference VP1 sequences included in this sequence

**Sample ID**	**GenBank Accession No.**	**Serotype**	**Topotype**	**Lineage**	**Sublineage**	**References**
O/BAN/BLRI/450.1/2018	MT316588.1	O	ME-SA	Ind-2001	Ind-2001e	https://doi.org/10.4314/ovj.v10i3.14
BAN/TA/Dh-299/2016 (O/ME-SA/Ind-2001/Ind-2001e)	KY077627.1	O	ME-SA	Ind-2001	Ind-2001e	https://doi.org/10.1111/tbed.12834
BAN/MG/Sa-294/2016 (O/ME-SA/Ind-2001/Ind-2001e)	KY077626.1	O	ME-SA	Ind-2001	Ind-2001e	https://doi.org/10.1111/tbed.12834

BAN/SI/Sh-234/2015 (O/ME-SA/Ind-2001/Ind-2001e)	KY077610.1	O	ME-SA	Ind-2001	Ind-2001e	https://doi.org/10.1111/tbed.12834
BAN/DI/Sa-252/2015 (O/ME-SA/Ind-2001/Ind-2001e)	KY077616.1	O	ME-SA	Ind-2001	Ind-2001e	https://doi.org/10.1111/tbed.12834
BAN/LK/Sa-248/2015 (O/ME-SA/Ind-2001/Ind-2001e)	KY077612.1	O	ME-SA	Ind-2001	Ind-2001e	https://doi.org/10.1111/tbed.12834
BAN/TG/Ba-268/2015 (O/ME-SA/Ind-2001/Ind-2001e)	KY077621.1	O	ME-SA	Ind-2001	Ind-2001e	https://doi.org/10.1111/tbed.12834
BAN/LK/Sa-249/2015 (O/ME-SA/Ind-2001/Ind-2001e)	KY077613.1	O	ME-SA	Ind-2001	Ind-2001e	https://doi.org/10.1111/tbed.12834
BAN/TA/Dh-301/2016 (O/ME-SA/Ind-2001/Ind-2001e)	MK088170.1	O	ME-SA	Ind-2001	Ind-2001e	https://doi.org/10.1111/tbed.12834
BAN/GO/Ka-236(Pig)/2015 (O/ME-SA/Ind-2001/Ind-2001e)	KX712091.1	O	ME-SA	Ind-2001	Ind-2001e	https://doi.org/10.1128/genomeA.01150-16

BAN/BO/Na-161/2013 (O/ME-SA/Ind-2001/Ind-2001BD2)	MK071699.1	O	ME-SA	Ind-2001	Ind-2001BD2	https://doi.org/10.1111/tbed.12834
BAN/BO/Na-162/2013 (O/ME-SA/Ind-2001/Ind-2001BD2)	KY077601.1	O	ME-SA	Ind-2001	Ind-2001BD2	https://doi.org/10.1111/tbed.12834
BAN LA Sa-137 2013 (O/ME-SA/Ind-2001/Ind-2001BD2)	KJ175182.1	O	ME-SA	Ind-2001	Ind-2001BD2	https://doi.org/10.1111/tbed.12834
O/KUW/3/97 (O/ME-SA/Ind-2001/Ind-2001a)	DQ164904.1	O	ME-SA	Ind-2001	Ind-2001a	https://doi.org/10.3201/eid1112.050908
O/UAE/7/97 (O/ME-SA/Ind-2001/Ind-2001a)	AJ318856.1	O	ME-SA	Ind-2001	Ind-2001a	https://doi.org/10.3201/eid1112.050908
O/OMN/7/2001 (O/ME-SA/Ind-2001/Ind-2001b)	DQ164941.1	O	ME-SA	Ind-2001	Ind-2001b	https://doi.org/10.3201/eid1112.050908
O/BAR/1/2001 (O/ME-SA/Ind-2001/Ind-2001b)	DQ164863.1	O	ME-SA	Ind-2001	Ind-2001b	https://doi.org/10.3201/eid1112.050908
O/IND187/2012 (O/ME-SA/Ind-2001/Ind-2001b)	KC506545.1	O	ME-SA	Ind-2001	Ind-2001b	https://doi.org/10.1016/j.meegid.2013.04.027
O/IND62/2011 (O/ME-SA/Ind-2001/Ind-2001b)	KC506543.1	O	ME-SA	Ind-2001	Ind-2001b	https://doi.org/10.1016/j.meegid.2013.04.027

UAE/4/2008 (O/ME-SA/Ind-2001/Ind-2001c)	KM921876.1	O	ME-SA	Ind-2001	Ind-2001c	https://doi.org/10.1111/tbed.12299
UAE/9/2009 (O/ME-SA/Ind-2001/Ind-2001c)	KJ606983.1	O	ME-SA	Ind-2001	Ind-2001c	https://doi.org/10.1186/1743-422X-11-136
O/IND/35/2009 (O/ME-SA/Ind-2001/Ind-2001c)	MN983157.1	O	ME-SA	Ind-2001	Ind-2001c	https://doi.org/10.1128%2FMRA.00287-20
BAN/1/2009 (O/ME-SA/Ind-2001/Ind-2001d)	HQ630676.1	O	ME-SA	Ind-2001	Ind-2001d	https://doi.org/10.1111/j.1865-1682.2011.01206.x
BAN/26/2009 (O/ME-SA/Ind-2001/Ind-2001d)	HQ630687.1	O	ME-SA	Ind-2001	Ind-2001d	https://doi.org/10.1111/j.1865-1682.2011.01206.x
BAN/25/2009 (O/ME-SA/Ind-2001/Ind-2001d)	HQ630686.1	O	ME-SA	Ind-2001	Ind-2001d	https://doi.org/10.1111/j.1865-1682.2011.01206.x
BAN/2/2009 (O/ME-SA/Ind-2001/Ind-2001d)	HQ630677.1	O	ME-SA	Ind-2001	Ind-2001d	https://doi.org/10.1111/j.1865-1682.2011.01206.x
BAN/9/2009 (O/ME-SA/Ind-2001/Ind-2001d)	HQ630681.1	O	ME-SA	Ind-2001	Ind-2001d	https://doi.org/10.1111/j.1865-1682.2011.01206.x

BD SI 6 2013 (O/ME-SA/Ind-2001/Ind-2001d)	KT037120.1	O	ME-SA	Ind-2001	Ind-2001d	https://doi.org/10.1111/tbed.12834
BAN LA Du-135 2013 (O/ME-SA/Ind-2001/Ind-2001d)	KJ175181.1	O	ME-SA	Ind-2001	Ind-2001d	https://doi.org/10.1111/tbed.12834
BAN TA Dh-184 2013 (O/ME-SA/Ind-2001/Ind-2001d)	KJ175184.1	O	ME-SA	Ind-2001	Ind-2001d	https://doi.org/10.1111/tbed.12834
BAN/GA/Kk-191/2013 (O/ME-SA/Ind-2001/Ind-2001d)	KY077602.1	O	ME-SA	Ind-2001	Ind-2001d	https://doi.org/10.1111/tbed.12834
BAN/GA/Ka-215/2015 (O/ME-SA/Ind-2001/Ind-2001d)	KY077607.1	O	ME-SA	Ind-2001	Ind-2001d	https://doi.org/10.1111/tbed.12834
BAN/PA/Ch-228/2015 (O/ME-SA/Ind-2001/Ind-2001d)	KY077609.1	O	ME-SA	Ind-2001	Ind-2001d	https://doi.org/10.1111/tbed.12834
IC120/2018	MT936103.1	O	ME-SA	Ind-2001	Ind-2001e	https://doi.org/10.1111/tbed.13954
IC70/2018	MT936099.1	O	ME-SA	Ind-2001	Ind-2001e	https://doi.org/10.1111/tbed.13954
BHU/2/2018	MT276847.1	O	ME-SA	Ind-2001	Ind-2001e	https://doi.org/10.1111/tbed.13954

IC214/2018	MT936112.1	O	ME-SA	Ind-2001	Ind-2001e	https://doi.org/10.1111/tbed.13954
IC117/2018	MT936101.1	O	ME-SA	Ind-2001	Ind-2001e	https://doi.org/10.1111/tbed.13954
IC72/2018	MT936100.1	O	ME-SA	Ind-2001	Ind-2001e	https://doi.org/10.1111/tbed.13954
IC03/2018	MT936090.1	O	ME-SA	Ind-2001	Ind-2001e	https://doi.org/10.1111/tbed.13954
PD396/2018	MT919008.1	O	ME-SA	Ind-2001	Ind-2001e	https://doi.org/10.1111/tbed.13954
PD372/2018	MT919006.1	O	ME-SA	Ind-2001	Ind-2001e	https://doi.org/10.1111/tbed.13954
PD119/2015	MT909624.1	O	ME-SA	Ind-2001	Ind-2001e	https://doi.org/10.1111/tbed.13954
PD498/2017	MT918987.1	O	ME-SA	Ind-2001	Ind-2001e	https://doi.org/10.1111/tbed.13954
O/IND182/2011 (O/ME-SA/Ind-2011)	KC506553.1	O	ME-SA	Ind-2011		https://doi.org/10.1016/j.meegid.2013.04.027
O/IND91/2012 (O/ME-SA/Ind-2011)	KC506549.1	O	ME-SA	Ind-2011		https://doi.org/10.1016/j.meegid.2013.04.027

O/LAO/2/2006 (O/ME-SA/PanAsia)	EU667451.1	O	ME-SA	PanAsia		https://doi.org/10.1128/genomeA.01150-16
O/CAM/1/2008 (O/ME-SA/PanAsia)	HQ116174.1	O	ME-SA	PanAsia		https://doi.org/10.1016/j.meegid.2010.11.003
O/LAO/28/2003 (O/ME-SA/PanAsia)	DQ164916.1	O	ME-SA	PanAsia		https://doi.org/10.3201/eid1112.050908
UKG/35/2001 (O/ME-SA/PanAsia)	AJ539141.1	O	ME-SA	PanAsia		https://doi.org/10.1099/vir.0.18669-0
O/BHU/1/98 (O/ME-SA/PanAsia)	AJ318826.1	O	ME-SA	PanAsia		https://doi.org/10.3201/eid1112.050908
O/NEP/2/99 (O/ME-SA/PanAsia)	DQ164937.1	O	ME-SA	PanAsia		https://doi.org/10.3201/eid1112.050908
O/NEP/4/98 (O/ME-SA/PanAsia)	DQ164935.1	O	ME-SA	PanAsia		https://doi.org/10.3201/eid1112.050908
O/NEP/9/98 (O/ME-SA/PanAsia)	DQ164936.1	O	ME-SA	PanAsia		https://doi.org/10.3201/eid1112.050908
O/TAI/2/2003 (O/ME-SA/PanAsia)	DQ164980.1	O	ME-SA	PanAsia		https://doi.org/10.3201/eid1112.050908
O/TUR/8/2000 (O/ME-SA/PanAsia)	DQ164984.1	O	ME-SA	PanAsia		https://doi.org/10.3201/eid1112.050908

O/VIT/8/2004 (O/ME-SA/PanAsia)	HQ116275.1	O	ME-SA	PanAsia		https://doi.org/10.1016/j.meegid.2010.11.003
TUR/257/2008 (PanAsia-2/TER-08)	MW085854.1	O	ME-SA	PanAsia-2	TER-08	https://doi.org/10.1093/molbev/msab172
Ber-Sheva-07 (PanAsia-2/TER-08)	HM561393.1	O	ME-SA	PanAsia-2	TER-08	https://doi.org/10.1016/j.vetmic.2010.07.004
Bet-Sharim-07 (PanAsia-2/TER-08)	HM561395.1	O	ME-SA	PanAsia-2	TER-08	https://doi.org/10.1016/j.vetmic.2010.07.004
Eilot-07 (PanAsia-2/TER-08)	HM561394.1	O	ME-SA	PanAsia-2	TER-08	https://doi.org/10.1016/j.vetmic.2010.07.004
Neve-Ur-07 (PanAsia-2/TER-08)	HM561402.1	O	ME-SA	PanAsia-2	TER-08	https://doi.org/10.1016/j.vetmic.2010.07.004
IRN/21/2016 (PanAsia-2/QOM-15)	MT443399.1	O	ME-SA	PanAsia-2	QOM-15	https://doi.org/10.1093/molbev/msab172
IRN/25/2016 (PanAsia-2/QOM-15)	MT443401.1	O	ME-SA	PanAsia-2	QOM-15	https://doi.org/10.1093/molbev/msab172
IRN/13/2016 (PanAsia-2/QOM-15)	MT443393.1	O	ME-SA	PanAsia-2	QOM-15	https://doi.org/10.1093/molbev/msab172

IRN/16/2015 (PanAsia-2/QOM-15)	MT443387.1	O	ME-SA	PanAsia-2	QOM-15	https://doi.org/10.1093/molbev/msab172
IRN/11/2018 (PanAsia-2/QOM-15)	MT443416.1	O	ME-SA	PanAsia-2	QOM-15	https://doi.org/10.1093/molbev/msab172
IRN/16/2018 (PanAsia-2/QOM-15)	MT443420.1	O	ME-SA	PanAsia-2	QOM-15	https://doi.org/10.1093/molbev/msab172
TUR/4/2010 (PanAsia-2/SAN-09)	MN276046.1	O	ME-SA	PanAsia-2	SAN-09	https://doi.org/10.1111/tbed.13502
TUR/264/2009 (PanAsia-2/SAN-09)	MW085870.1	O	ME-SA	PanAsia-2	SAN-09	https://doi.org/10.1093/molbev/msab172
TUR/9/2010 (PanAsia-2/SAN-09)	MT443830.1	O	ME-SA	PanAsia-2	SAN-09	https://doi.org/10.1093/molbev/msab172
NEP/1/2015 (PanAsia-2/KAT-15)	MN276045.1	O	ME-SA	PanAsia-2	KAT-15	https://doi.org/10.1111/tbed.13502
ISR/2/2018 (PanAsia-2/QOM-15)	MT443489.1	O	ME-SA	PanAsia-2	QOM-15	https://doi.org/10.1093/molbev/msab172
ISR/4/2018 (PanAsia-2/QOM-15)	MT443491.1	O	ME-SA	PanAsia-2	QOM-15	https://doi.org/10.1093/molbev/msab172
KUW/1/2016 (PanAsia-2/QOM-15)	MT443517.1	O	ME-SA	PanAsia-2	QOM-15	https://doi.org/10.1093/molbev/msab172
KUW/4/2016 (PanAsia-2/QOM-15)	MT443519.1	O	ME-SA	PanAsia-2	QOM-15	https://doi.org/10.1093/molbev/msab172

PAK/32/2015 (PanAsia-2/QOM-15)	MT443734.1	O	ME-SA	PanAsia-2	QOM-15	https://doi.org/10.1093/molbev/msab172
PAK/34/2015 (PanAsia-2/QOM-15)	MT443735.1	O	ME-SA	PanAsia-2	QOM-15	https://doi.org/10.1093/molbev/msab172
TUR/276/2017 (PanAsia-2/QOM-15)	MT442857.1	O	ME-SA	PanAsia-2	QOM-15	https://doi.org/10.1093/molbev/msab172
AFG/20/2010 (PanAsia-2/PUN-10)	MT442857.1	O	ME-SA	PanAsia-2	PUN-10	https://doi.org/10.1093/molbev/msab172
PAK/35/2010 (PanAsia-2/PUN-10)	MT443598.1	O	ME-SA	PanAsia-2	PUN-10	https://doi.org/10.1093/molbev/msab172
PAK/19/2010 (PanAsia-2/PUN-10)	MT443589.1	O	ME-SA	PanAsia-2	PUN-10	https://doi.org/10.1093/molbev/msab172
PAK/16/2010 (PanAsia-2/PUN-10)	KY091285.1	O	ME-SA	PanAsia-2	PUN-10	https://doi.org/10.20506/rst.35.3.2565
PAK/37/2010 (PanAsia-2/PUN-10)	MT443600.1	O	ME-SA	PanAsia-2	PUN-10	https://doi.org/10.1093/molbev/msab172
PAK/20/2010 (PanAsia-2/PUN-10)	MT443590.1	O	ME-SA	PanAsia-2	PUN-10	https://doi.org/10.1093/molbev/msab172
PAK/17/2010 (PanAsia-2/PUN-10)	MT443587.1	O	ME-SA	PanAsia-2	PUN-10	https://doi.org/10.1093/molbev/msab172
PAK/18/2010 (PanAsia-2/PUN-10)	MT443588.1	O	ME-SA	PanAsia-2	PUN-10	https://doi.org/10.1093/molbev/msab172

SAU/1/2009 (PanAsia-2/FAR-09)	MT443788.1	O	ME-SA	PanAsia-2	FAR-09	https://doi.org/10.1093/molbev/msab172
IRN/54/2009 (PanAsia-2/FAR-09)	MT443173.1	O	ME-SA	PanAsia-2	FAR-09	https://doi.org/10.1093/molbev/msab172
IRN/71/2009 (PanAsia-2/FAR-09)	MT443184.1	O	ME-SA	PanAsia-2	FAR-09	https://doi.org/10.1093/molbev/msab172
IRN/76/2009 (PanAsia-2/FAR-09)	MT443186.1	O	ME-SA	PanAsia-2	FAR-09	https://doi.org/10.1093/molbev/msab172
IRN/8/2010 (PanAsia-2/FAR-09)	MT443201.1	O	ME-SA	PanAsia-2	FAR-09	https://doi.org/10.1093/molbev/msab172
IRN/7/2015 (PanAsia-2/FAR-09)	MT443385.1	O	ME-SA	PanAsia-2	FAR-09	https://doi.org/10.1093/molbev/msab172
ISR/1/2014 (PanAsia-2/FAR-09)	MT443482.1	O	ME-SA	PanAsia-2	FAR-09	https://doi.org/10.1093/molbev/msab172
TUR/10/2010 (PanAsia-2/FAR-09)	MT443831.1	O	ME-SA	PanAsia-2	FAR-09	https://doi.org/10.1093/molbev/msab172
TUR/12/2013 (PanAsia-2/FAR-09)	MT443859.1	O	ME-SA	PanAsia-2	FAR-09	https://doi.org/10.1093/molbev/msab172
TUR/27/2013 (PanAsia-2/FAR-09)	MT443864.1	O	ME-SA	PanAsia-2	FAR-09	https://doi.org/10.1093/molbev/msab172
TUR/1156/2013 (PanAsia-2/FAR-09)	MW085896.1	O	ME-SA	PanAsia-2	FAR-09	https://doi.org/10.1093/molbev/msab172

TUR/33/2014 (PanAsia-2/FAR-09)	MT443881.1	O	ME-SA	PanAsia-2	FAR-09	https://doi.org/10.1093/molbev/msab172
TUR/17/2015 (PanAsia-2/FAR-09)	MT443884.1	O	ME-SA	PanAsia-2	FAR-09	https://doi.org/10.1093/molbev/msab172
TUR/10/2015 (PanAsia-2/FAR-09)	MT443883.1	O	ME-SA	PanAsia-2	FAR-09	https://doi.org/10.1093/molbev/msab172
AFG/19/2009 (PanAsia-2/BAL-09)	MT442823.1	O	ME-SA	PanAsia-2	BAL-09	https://doi.org/10.1093/molbev/msab172
AFG/27/2009 (PanAsia-2/BAL-09)	MT442828.1	O	ME-SA	PanAsia-2	BAL-09	https://doi.org/10.1093/molbev/msab172
AFG/66/2009 (PanAsia-2/BAL-09)	MT442847.1	O	ME-SA	PanAsia-2	BAL-09	https://doi.org/10.1093/molbev/msab172
AFG/23/2010 (PanAsia-2/BAL-09)	MT442860.1	O	ME-SA	PanAsia-2	BAL-09	https://doi.org/10.1093/molbev/msab172
AFG/53/2010 (PanAsia-2/BAL-09)	MT442878.1	O	ME-SA	PanAsia-2	BAL-09	https://doi.org/10.1093/molbev/msab172
IRN/18/2010 (PanAsia-2/BAL-09)	KY091283.1	O	ME-SA	PanAsia-2	BAL-09	https://doi.org/10.20506/rst.35.3.2565
AFG/26/2010 (PanAsia-2/BAL-09)	MT442863.1	O	ME-SA	PanAsia-2	BAL-09	https://doi.org/10.1093/molbev/msab172
AFG/2/2010 (PanAsia-2/BAL-09)	MT442850.1	O	ME-SA	PanAsia-2	BAL-09	https://doi.org/10.1093/molbev/msab172

AFG/38/2009 (PanAsia-2/BAL-09)	MT442838.1	O	ME-SA	PanAsia-2	BAL-09	https://doi.org/10.1093/molbev/msab172
PD57/2018	MT918999.1	O	ME-SA	SA-2018		https://doi.org/10.1111/tbed.13954
PD42/2018	MT918995.1	O	ME-SA	SA-2018		https://doi.org/10.1111/tbed.13954
PD295/2018	MT919004.1	O	ME-SA	SA-2018		https://doi.org/10.1111/tbed.13954
PD325/2018	MT919005.1	O	ME-SA	SA-2018		https://doi.org/10.1111/tbed.13954
PD45/2018	MT918997.1	O	ME-SA	SA-2018		https://doi.org/10.1111/tbed.13954
IC231/2018	MT936115.1	O	ME-SA	SA-2018		https://doi.org/10.1111/tbed.13954
IC207/2018	MT936111.1	O	ME-SA	SA-2018		https://doi.org/10.1111/tbed.13954
IC218/2018	MT936113.1	O	ME-SA	SA-2018		https://doi.org/10.1111/tbed.13954
PD38/2018	MT918994.1	O	ME-SA	SA-2018		https://doi.org/10.1111/tbed.13954
IC202/2018	MT936110.1	O	ME-SA	SA-2018		https://doi.org/10.1111/tbed.13954

PD224/2018	MT919002.1	O	ME-SA	SA-2018		https://doi.org/10.1111/tbed.13954
BD BAU ML1 2013	KT960948.1	O	ME-SA	PanAsia-2		https://doi.org/10.5455/javar.2020.g429
BD BAU ML2 2013	KT982203.1	O	ME-SA	PanAsia-2		https://doi.org/10.5455/javar.2020.g429
O/BAN/BLRI/450.2/2018	MT316587.1	O	ME-SA	Uncharacterized		https://doi.org/10.4314/ovj.v10i3.14
O/IND23/2012	KC506439.1	O	ME-SA	Uncharacterized		https://doi.org/10.1016/j.meegid.2013.04.027
O/IND172/2011	KC506438.1	O	ME-SA	Uncharacterized		https://doi.org/10.1016/j.meegid.2013.04.027
O/IND35/2011	KC506441.1	O	ME-SA	Uncharacterized		https://doi.org/10.1016/j.meegid.2013.04.027
O/IND52/2011	KC506442.1	O	ME-SA	Uncharacterized		https://doi.org/10.1016/j.meegid.2013.04.027
O/IND56/2011	KC506443.1	O	ME-SA	Uncharacterized		https://doi.org/10.1016/j.meegid.2013.04.027

BD SI 5 2013	KT037118.1	O	ME-SA	Uncharacterized		https://doi.org/10.1111/tbed.12834
UGA/5/96 (O/EA-1)	AJ296327.1	O	EA-1	EA-1		https://doi.org/10.3201/eid1112.050908
O/KEN/5/2002 (O/EA-2)	DQ165073.1	O	EA-2	EA-2		https://doi.org/10.3201/eid1112.050908
TAN/2/2004 (O/EA-2)	KF561679.1	O	EA-2	EA-2		https://doi.org/10.1111/tbed.12200
O/Fayoum/EGY/2014 (O/EA-3)	KR261670.1	O	EA-3	EA-3		https://doi.org/10.14202%2Fvetworld.2019.190-197
O/Qaliubia/EGY/2013 (O/EA-3)	KR261668.1	O	EA-3	EA-3		https://doi.org/10.14202%2Fvetworld.2019.190-197
ETH/60/2005 (O/EA-4)	FJ798143.1	O	EA-4	EA-4		https://doi.org/10.3201/eid1509.090091
CAR/16/2000 (O/WA)	HM211080.1	O	WA	WA		https://doi.org/10.1111/j.1865-1682.2010.01151.x
O/PHI/7/96 (O/CATHAY)	AJ294926.1	O	CATHAY	CATHAY		https://doi.org/10.3201/eid1112.050908

O/HKN/6/83 (O/CATHAY)	AJ294919.1	O	CATHAY	CATHAY		https://doi.org/10.3201/eid1112.050908
O/Corrientes/Arg/06 (O/EURO-SA)	DQ834727.1	O	EURO-SA	EURO-SA		https://doi.org/10.1016/j.virusres.2006.09.006
O/Chuquisaca/Bol/03 (O/EURO-SA)	DQ834712.1	O	EURO-SA	EURO-SA		https://doi.org/10.1016/j.virusres.2006.09.006
O/HongKong/P424/2011 (O/SEA)	JF968193.1	O	SEA	SEA		http://dx.doi.org/10.1016/j.vetmic.2012.03.016
O/HongKong/P404/2010 (O/SEA)	JF968186.1	O	SEA	SEA		http://dx.doi.org/10.1016/j.vetmic.2012.03.016
ISA/8/83 (O/ISA-1)	AJ303503.1	O	ISA-1	ISA-1		https://doi.org/10.3201/eid1112.050908
JAV/5/72 (O/ISA-2)	AJ303509.1	O	ISA-2	ISA-2		https://doi.org/10.3201/eid1112.050908

❖ **<u>Supplementary Figures:</u>**

❖ Molecular characterization of MYMBD21

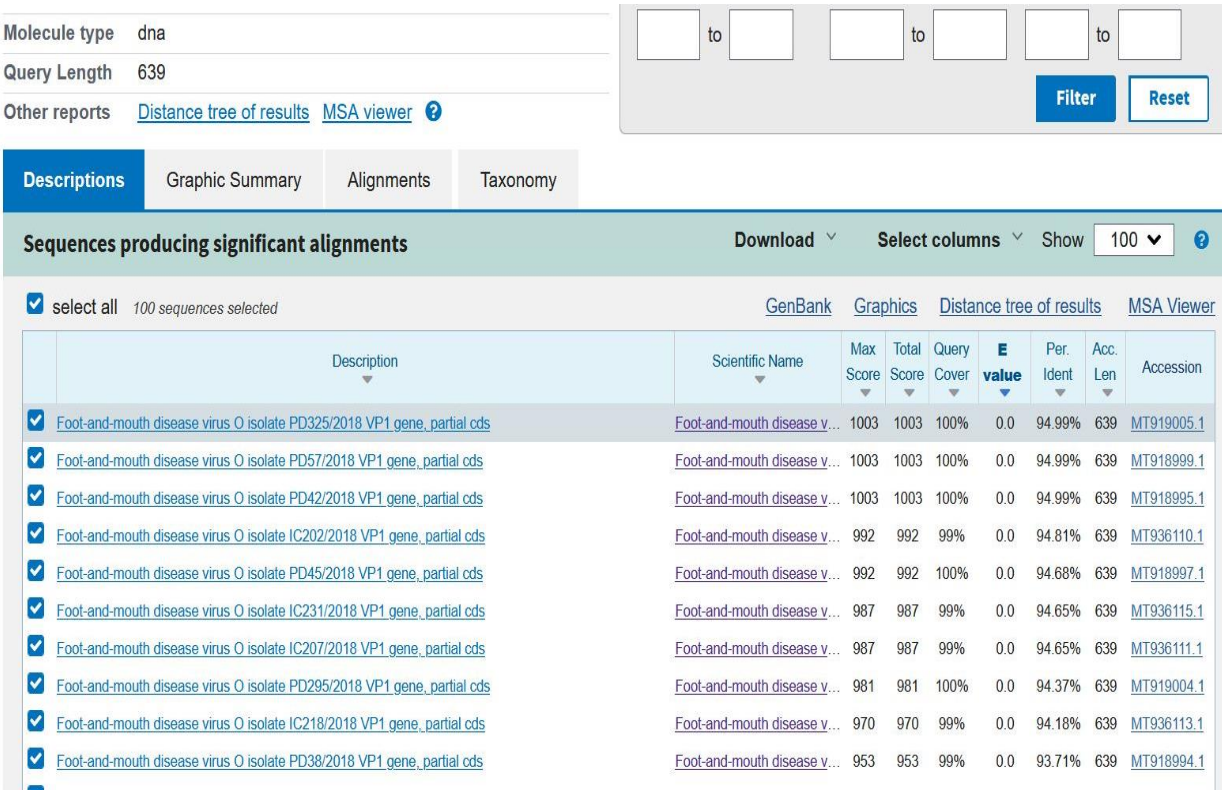

Molecule type	dna
Query Length	639
Other reports	Distance tree of results MSA viewer

Descriptions | Graphic Summary | Alignments | Taxonomy

Sequences producing significant alignments — Download — Select columns — Show 100

select all *100 sequences selected* — GenBank — Graphics — Distance tree of results — MSA Viewer

Description	Scientific Name	Max Score	Total Score	Query Cover	E value	Per. Ident	Acc. Len	Accession
Foot-and-mouth disease virus O isolate PD325/2018 VP1 gene, partial cds	Foot-and-mouth disease v...	1003	1003	100%	0.0	94.99%	639	MT919005.1
Foot-and-mouth disease virus O isolate PD57/2018 VP1 gene, partial cds	Foot-and-mouth disease v...	1003	1003	100%	0.0	94.99%	639	MT918999.1
Foot-and-mouth disease virus O isolate PD42/2018 VP1 gene, partial cds	Foot-and-mouth disease v...	1003	1003	100%	0.0	94.99%	639	MT918995.1
Foot-and-mouth disease virus O isolate IC202/2018 VP1 gene, partial cds	Foot-and-mouth disease v...	992	992	99%	0.0	94.81%	639	MT936110.1
Foot-and-mouth disease virus O isolate PD45/2018 VP1 gene, partial cds	Foot-and-mouth disease v...	992	992	100%	0.0	94.68%	639	MT918997.1
Foot-and-mouth disease virus O isolate IC231/2018 VP1 gene, partial cds	Foot-and-mouth disease v...	987	987	99%	0.0	94.65%	639	MT936115.1
Foot-and-mouth disease virus O isolate IC207/2018 VP1 gene, partial cds	Foot-and-mouth disease v...	987	987	99%	0.0	94.65%	639	MT936111.1
Foot-and-mouth disease virus O isolate PD295/2018 VP1 gene, partial cds	Foot-and-mouth disease v...	981	981	100%	0.0	94.37%	639	MT919004.1
Foot-and-mouth disease virus O isolate IC218/2018 VP1 gene, partial cds	Foot-and-mouth disease v...	970	970	99%	0.0	94.18%	639	MT936113.1
Foot-and-mouth disease virus O isolate PD38/2018 VP1 gene, partial cds	Foot-and-mouth disease v...	953	953	99%	0.0	93.71%	639	MT918994.1

Supplementary Figure S1 BLAST search results of VP1 of representative MYMBD21 isolate. The search result is showing ~94-95% identity with VP1 sequences of SA-2018 lineage reported in India in 2018.

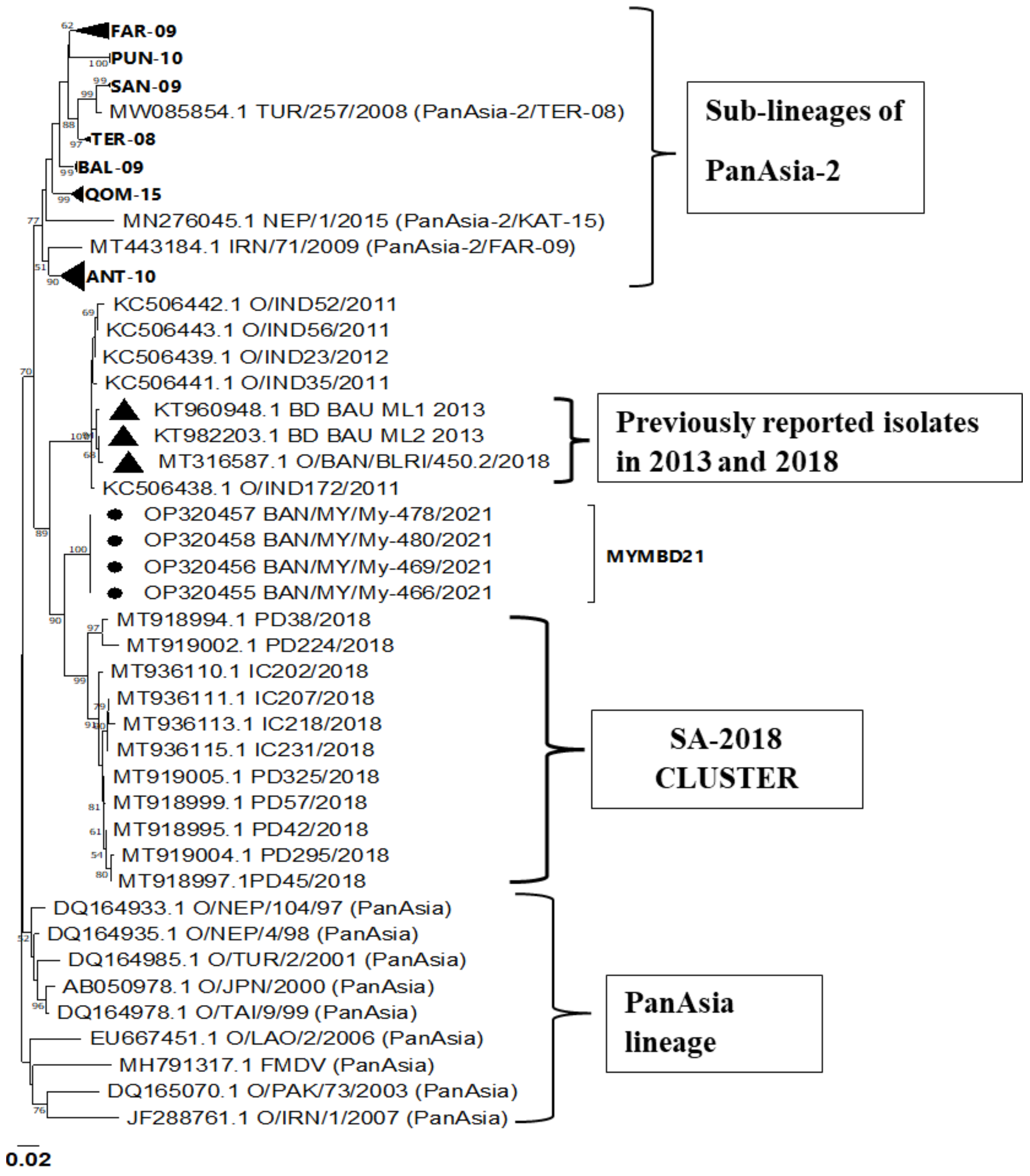

Supplementary Figure S2 Neighbor-joining phylogenetic tree showing distinct clade formed by MYMBD21 isolates from SA-2018 and uncategorized isolates reported from Bangladesh. Phylogenetic tree was generated using MEGA11[1].

In the phylogenetic tree, MYMBD21 formed a distinct clade from SA-2018 **(Figure S2)** and the divergence was 5-6% according to genetic distance analysis **(Table S3)** and BLAST tool (**Figure S1)** which confirms that MYMBD21 is a novel sub-lineage that belongs to SA-2018 lineage. Again, the lowest genetic distance (0.062) between SA-2018 and MYMBD21 was lower than the distance between other established lineages suggesting that MYMBD21 belongs to the SA-2018 lineage (**Table S3).**

MYMBD21 isolates also showed 8% divergence from previously circulating Bangladeshi isolates which indicates these isolates do not fall under the same lineage and MYMBD21 is unique for Bangladesh **(Figure S4).**

❖ Mutational Analysis

➢ Variations in the VP1 nucleotide sequence of MYMBD21 with SA-2018:

MYMBD21 vs SA-2018

```
Range 1: 1 to 639  Graphics                                   ▼ Next Match ▲

NW Score        Identities            Gaps              Strand
1123            608/639(95%)          0/639(0%)         Plus/Plus

Query  1    ACAACCTCCACAGGTGAGTCGGCTGACCCCGTGACCGCCACCGTTGAGAACTACGGGGGC  60
Sbjct  1    ..........................T........T...........A........A...  60

Query  61   GAAACACAGGTCCAGAGACGTCAGCACACGGACGTTTCTTTCATATTGGACAGATTTGTG  120
Sbjct  61   ..G.................C..............C.......................A  120

Query  121  AAAGTGACACCAAAAGACCAAATCAATGTATTGGACCTGATGCAAACCCCCGCTCACACT  180
Sbjct  121  .......................T......C...................T.........  180

Query  181  TTGGTGGGTGCCCTTCTTCGCACCGCTACCTACTACTTCGCAGATTTAGAGGTGGCAGTG  240
Sbjct  181  .................C........C.................................  240

Query  241  AAACACGAGGGGGACCTCACCTGGGTCCCGAACGGAGCACCCGAAAAAGCCTTGGACAAC  300
Sbjct  241  .....T......A......................G..G.....................  300

Query  301  ACCACTAATCCAACGGCTTACCACAAGGCACCACTCACCCGACTTGCACTGCCGTACACG  360
Sbjct  301  .....C..........................G...........................  360

Query  361  GCACCACACCGTGTCTTGGCTACTGTCTACAACGGGAACTGCAAGTACGGCGAGAGCCGC  420
Sbjct  361  .....C.........A..........T...............................A.  420

Query  421  GCAACTAGTGTGAGAGGTGACCTGCAAGTGTTGGCCCAGAAGGCGGCAAGGACGCTGCCT  480
Sbjct  421  ........C...................................A...............  480

Query  481  ACCTCCTTTAACTATGGTGCCATCAAAGCTACTCGGGTGACTGAACTGCTTTACCGCATG  540
Sbjct  481  ........C.....C......................................T......  540

Query  541  AAGAGGGCTGAAACATACTGCCCTCGGCCTCTTCTGGCCATCCACCCGAGTGAAGCTAGA  600
Sbjct  541  .................................T..........................  600

Query  601  CACAAACAGAAGATTGTGGCACCTGTGAAACAACTTCTG  639
Sbjct  601  ........A....................G.........  639
```

Supplementary Figure S3 Nucleotide changes of MYMBD21 against SA-2018 consensus VP1 sequences (5% divergence) calculated using Needleman-Wunsch algorithm [7] in BLAST global alignment tool [Subject=MYMBD21; Query: SA-2018]

➢ **Variations in the VP1 nucleotide sequence of MYMBD21 with uncategorized Bangladeshi isolates:**

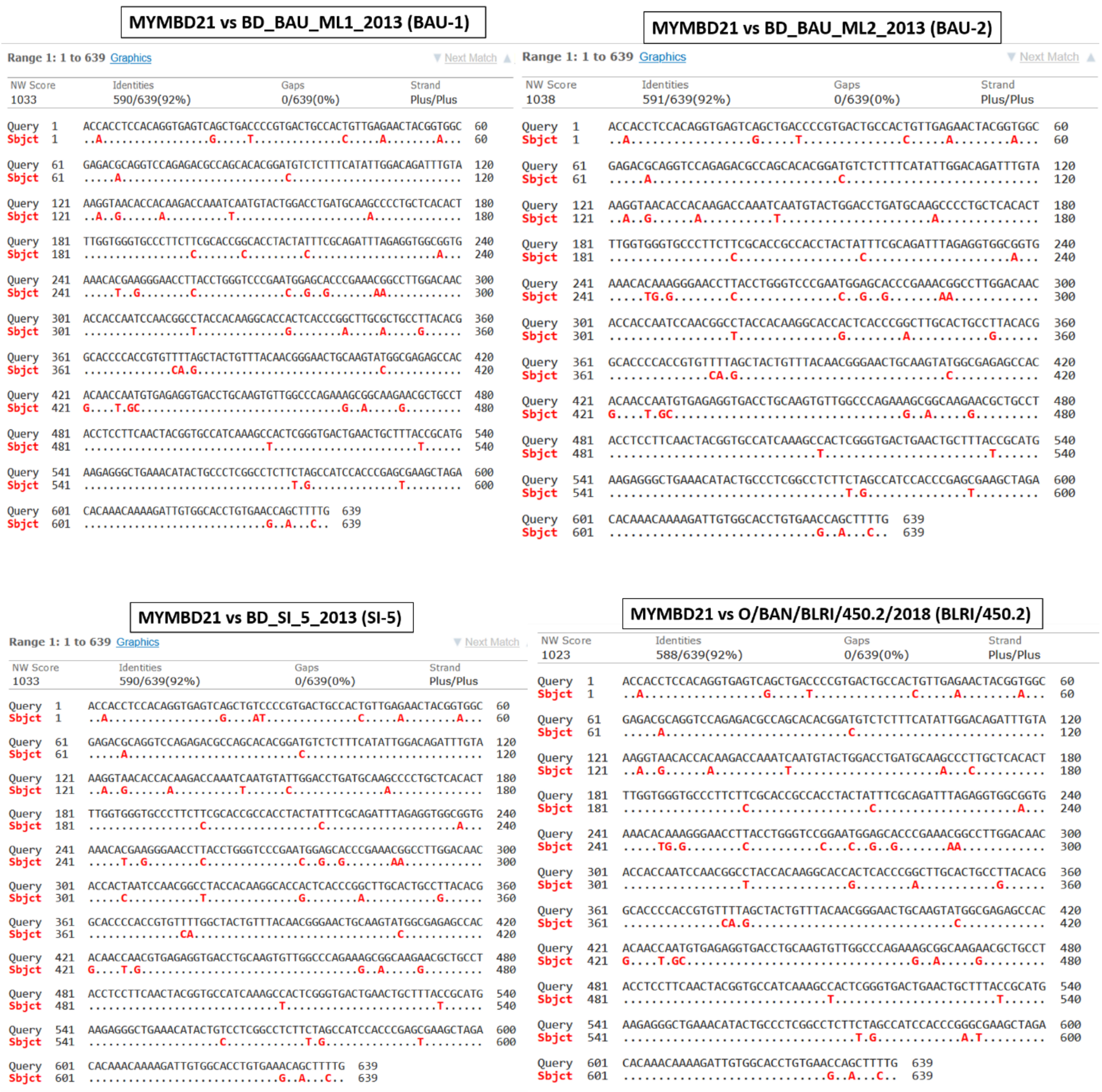

Supplementary Figure S4 Nucleotide changes of VP1 of MYMBD21 against uncharacterized Bangladeshi isolates (BD_BAU_ML1_2013; BD_BAU_ML2_2013; BD_SI_5_2013; O/BAN/BLRI/450.2/2018) showing 92%identity or 8% divergence calculated using Needleman-Wunsch algorithm [7] in BLAST global alignment tool.

[Subject=MYMBD21; Query: BAU-1; BAU-2, SI-5; BLRI/450.2]

Amino acid variations

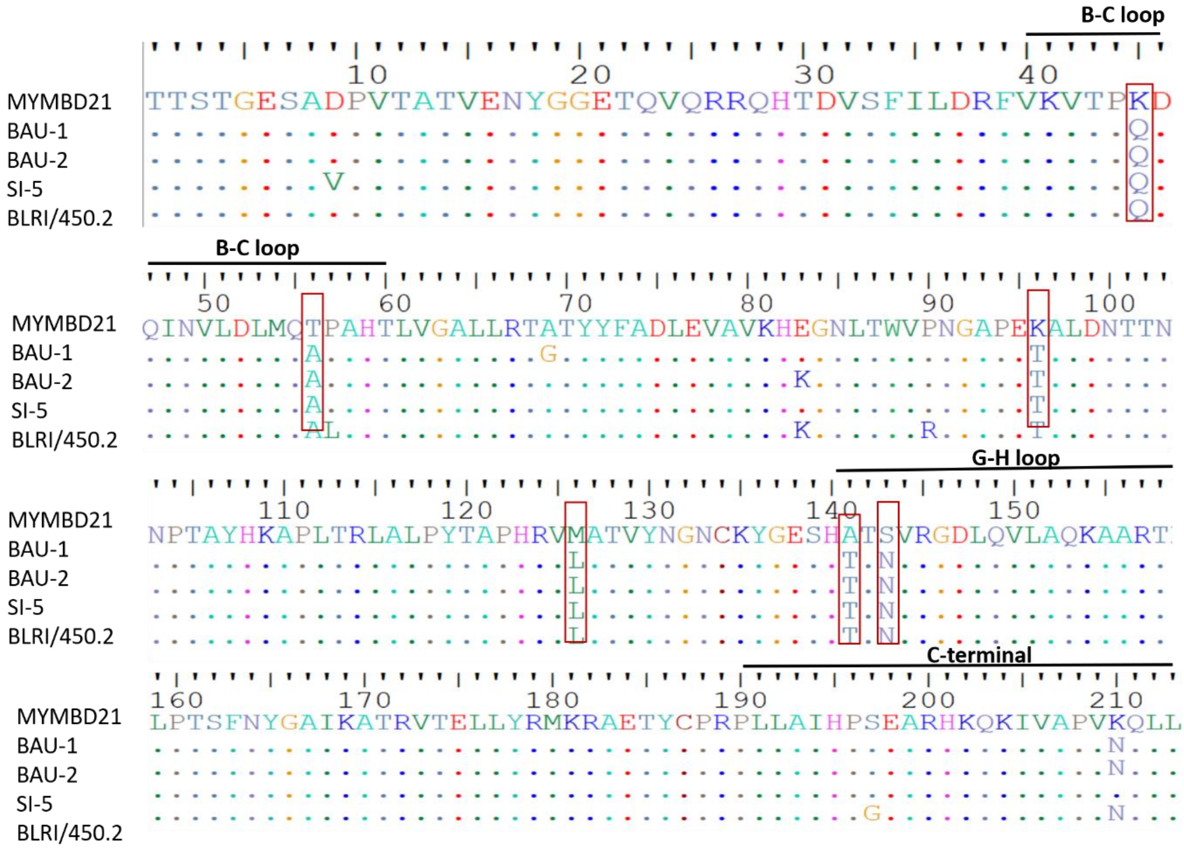

Supplementary Figure S5 Amino acid variations against MYMBD21 against uncharacterized Bangladeshi isolates that were reported during 2013 and 2018. VP1 amino acid sequences were aligned and viewed using BioEdit [8]

B-C loop:40-60 amino acid; G-H loop:130-160 amino acid; Cterminal:190-213 amino acid

[BAU-1: BD_BAU_ML1_2013; BAU-2: BD_BAU_ML1_2013; SI-5:BD_SI_5_2013; BLRI/450.2:O/BAN/BLRI/450.2/2018]

❖ Comparison with vaccine strains

➢ Variations in the VP1 nucleotide sequence of MYMBD21 with both current field vaccine and proposed local vaccine strains:

```
Range 1: 1 to 639  Graphics                                       ▼ Next Match

NW Score        Identities            Gaps              Strand
908             565/639(88%)          0/639(0%)         Plus/Plus

Query  1    ACCACCTCCACAGGTGAGTCAGCTGACCCCGTGACCGCCACTGTTGAAAACTACGGCGGT  60
Sbjct  1    ..A.................G.....T........T.....C..............A..C  60

Query  61   GAGACACAGGTCCAGAGGCGCCAACACACGGACGTCTCATTCATTTTGGACAGATTTGTA  120
Sbjct  61   .................A.....G..............T.....A...............  120

Query  121  AAAGTGACGCCAAAAGACCAAATTAATGTACTGGACCTGATGCAAACCCCCGCTCACACT  180
Sbjct  121  ........A.........................................T.........  180

Query  181  CTGGTGGGAGCGCTCCTTCGTACTGCCACTTACTATTTCGCTGACTTAGAAGTGGCAGTG  240
Sbjct  181  T.......T..C..T..C..C..C.....C.....C.....A..T.....G.........  240

Query  241  AAATACGAGGGAAACCTCACTTGGGTCCCGAATGGGGCGCCTGAAAACGCGTTGGATAAC  300
Sbjct  241  ...C.T.....G........C...........C........C.....A..C.....C...  300

Query  301  ACCACCAACCCAACGGCATACCACAAGGCACCACTCACCCGGCTTGCATTGCCGTACACG  360
Sbjct  301  ........T........T..............G........A......C...........  360

Query  361  GCACCACAACGTGTGTTGGCAACCGTTTACAACGGGAACTGCAAGTACGGTGATGGTTCG  420
Sbjct  361  .....C..C....CA.....T..T..........................C..GA.CCAC  420

Query  421  GTGACCAACATAAGAGGTGACCTACAAGTGTTGGCCCAGAAGGCGGCGAGAACGCTGCCT  480
Sbjct  421  .CA..T.G.G.G...........G....................A..A..G.........  480

Query  481  ACCTCCTTCAACTACGGTGCCATCAAAGCTACTCGGGTGACTGAACTGCTTTACCGCATG  540
Sbjct  481  .....................................................T......  540

Query  541  AAGAGGGCTGAGACGTACTGCCCCCGGCCTCTTTTGGCCATTCACCCGAACGAGGCCAGA  600
Sbjct  541  ...........A..A........T.................C.......GT..A..T...  600

Query  601  CACAAACAGAAGATTGTGGCACCTGTGAAGCAGCTCCTG  639
Sbjct  601  ........A.......................A..T...  639
```

Supplementary Figure S6 Nucleotide changes of MYMBD21 against current field vaccine strain (O/India/R2/75) showing 88% VP1 nucleotide identity that was calculated using Needleman-Wunsch algorithm [7] in BLAST global alignment tool. [Subject=MYMBD21; Query: vaccine strain]

Range 1: 1 to 639 Graphics   Next Match

NW Score	Identities	Gaps	Strand
878	559/639(87%)	0/639(0%)	Plus/Plus

```
Query  1    ACCACCTCCACAGGTGAGTCCGCTGATCCCGTGACCACCACCGTTGAGAACTACGGTGGA  60
Sbjct  1    ..A.................G..............TG..........A........A..C  60

Query  61   GAGACACAGGTCCAGAGACGTCAACACACCGACGTTTCTTTCATTTTGGACAGATTTGTG  120
Sbjct  61   ....................C..G.....G.....C........A..............A  120

Query  121  AAAGTAATACCGAAAGACCAAATCAATGTGTTGGACCTGATGCAAACCCCTGCTCACACT  180
Sbjct  121  .....G.C...A...........T.....AC.............................  180

Query  181  TTGGTAGGCGCACTCCTCCGCACCGCCACTTACTACTTCGCAGACCTAGAAGTGGCAGTG  240
Sbjct  181  .....G..T..C..T..............C..............TT....G.........  240

Query  241  AAGCACGAGGGCAACCTCACCTGGGTCCCGAACGGGGCGCCCGAGGCGGCGCTGGACAAC  300
Sbjct  241  ..A..T.....G................................AAAA..CT........  300

Query  301  ACCACCAACCCAACGGCCTACCACAAGGCACCGCTCACCCGTCTTGCTCTGCCTTACACA  360
Sbjct  301  ........T........T.......................A.....A.....G.....G  360

Query  361  GCACCACACCGTGTTCTGGCTACTGTTTACAACGGGAACTGCAAGTATGGCAAGGGCGCT  420
Sbjct  361  .....C........CA...............................C...G..A..CAC  420

Query  421  GTGACCAACGTGAGGGGTGACTTGCAAGTGTTGGCTCAGAAGGCAGCAAGAACGCTGCCC  480
Sbjct  421  .CA..T.G......A......C.............C..............G........T  480

Query  481  ACCTCCTTTAACTACGGTGCCATCAAGGCTACCCGGGTGACTGAACTGCTTTACCGCATG  540
Sbjct  481  ........C.................A.....T....................T......  540

Query  541  AAGAGGGCCGAAACATACTGCCCTCGGCCTCTGCTGGCCATTCACCCGGAACAAGCCAGA  600
Sbjct  541  ........T.......................TT.......C......AGTG....T...  600

Query  601  CACAAGCAGAAGATTGTGGCACCTGTGAAACAGTTGTTG  639
Sbjct  601  .....A..A....................G..AC.TC..  639
```

Supplementary Figure S7: Nucleotide changes of MYMBD21 against proposed local vaccine strain (BAN/TA/Dh-301/2016) showing 87% VP1 nucleotide identity which was calculated using Needleman-Wunsch algorithm [7] in BLAST global alignment tool. [Subject=MYMBD21; Query: vaccine strain]

❖ Amino acid variations with vaccine strains

Range 1: 1 to 213 Graphics   ▼ Next Match

NW Score	Identities	Positives	Gaps
1058	202/213(95%)	208/213(97%)	0/213(0%)

```
Query  1    TTSTGESADPVTATVENYGGETQVQRRQHTDVSFILDRFVKVTPKDQINVLDLMQTPAHT  60
Sbjct  1    ............................................................  60

Query  61   LVGALLRTATYYFADLEVAVKYEGNLTWVPNGAPENALDNTTNPTAYHKAPLTRLALPYT  120
Sbjct  61   .....................H.............K........................  120

Query  121  APQRVLATVYNGNCKYGDGSVTNIRGDLQVLAQKAARTLPTSFNYGAIKATRVTELLYRM  180
Sbjct  121  ..H..M...........ESHA.SV....................................  180

Query  181  KRAETYCPRPLLAIHPNEARHKQKIVAPVKQLL  213
Sbjct  181  ................S................  213
```

Supplementary Figure S8 Comparison of VP1 amino acid sequence between MYMBD21 (Subject) and current field vaccine strain (Query) showing 95% identity which was calculated using Needleman-Wunsch algorithm [7] in BLAST global alignment tool.

Range 1: 1 to 213 Graphics   ▼ Next Match

NW Score	Identities	Positives	Gaps
1056	202/213(95%)	206/213(96%)	0/213(0%)

```
Query  1    TTSTGESADPVTTTVENYGGETQVQRRQHTDVSFILDRFVKVIPKDQINVLDLMQTPAHT  60
Sbjct  1    ............A.............................T.................  60

Query  61   LVGALLRTATYYFADLEVAVKHEGNLTWVPNGAPEAALDNTTNPTAYHKAPLTRLALPYT  120
Sbjct  61   ...................................K........................  120

Query  121  APHRVLATVYNGNCKYGKGAVTNVRGDLQVLAQKAARTLPTSFNYGAIKATRVTELLYRM  180
Sbjct  121  .....M...........ESHA.S.....................................  180

Query  181  KRAETYCPRPLLAIHPEQARHKQKIVAPVKQLL  213
Sbjct  181  ................SE...............  213
```

Supplementary Figure S9 Comparison of VP1 amino acid sequence between MYMBD21 (Subject) and proposed local vaccine strain (Query) showing 95% identity which was calculated using Needleman-Wunsch algorithm [7] in BLAST global alignment tool.

❖ Validating the quality of protein structure

Ramachandran plot was developed using *Molprobityv4.4* [9] in SWISS-MODEL and Z-score was calculated using ProSA-web [10]

MYMBD21:

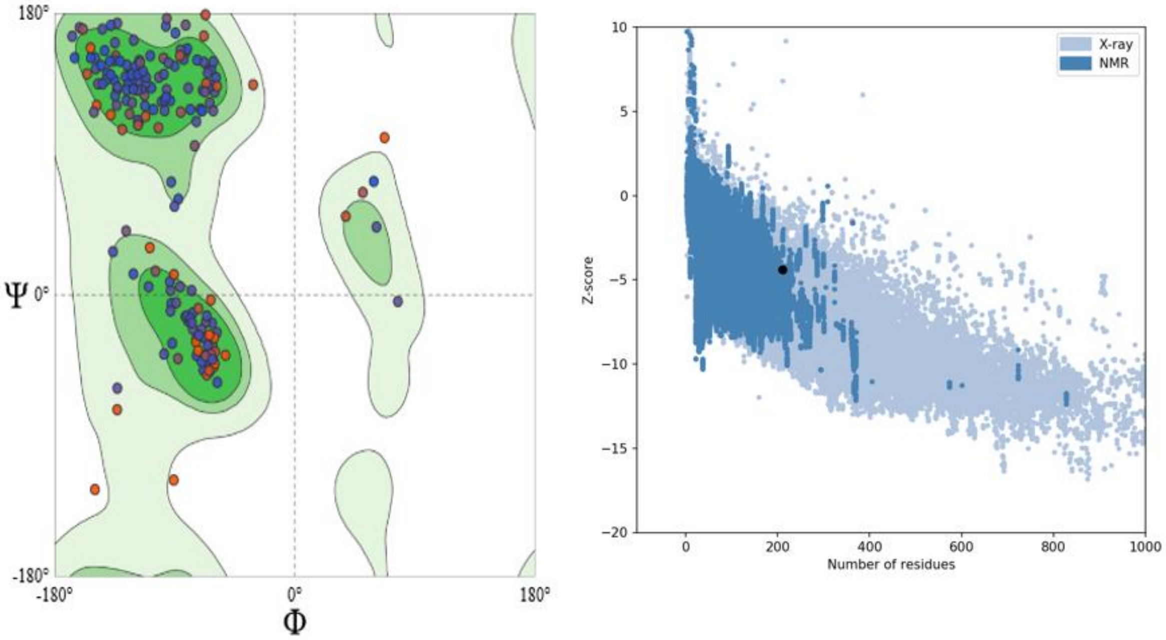

Supplementary Figure S10 Estimating the 3D model quality of consensus VP1 of MYMBD21 by Ramachandran plot and Z-score. In the plot, 94.23% of the amino acid residues were in the favored region with 1.44% outliers and Z-score was -4.41.

[The deep green and pale green colors represent the favored and allowed regions for an amino acid of the protein, respectively. On the X and Y axes, Φ (phi) and Ψ (psi) represented the torsion angles around alpha carbon to amine and carboxyl groups of different amino acids]

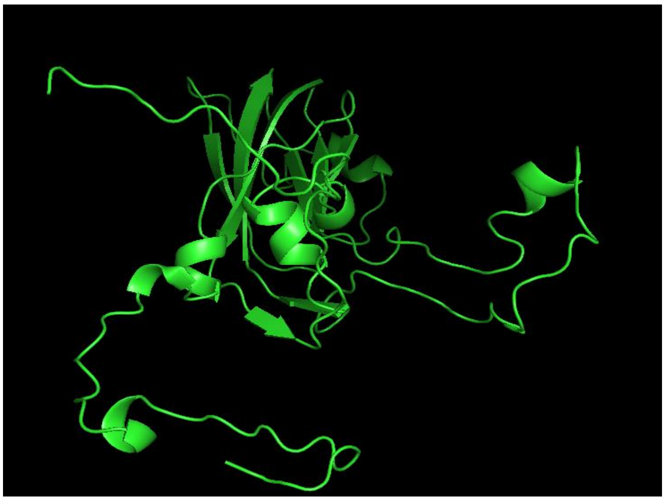

Supplementary Figure S11 3D model of consensus VP1 of MYMBD21 isolate generated using PyMOL[11] .

SA-2018:

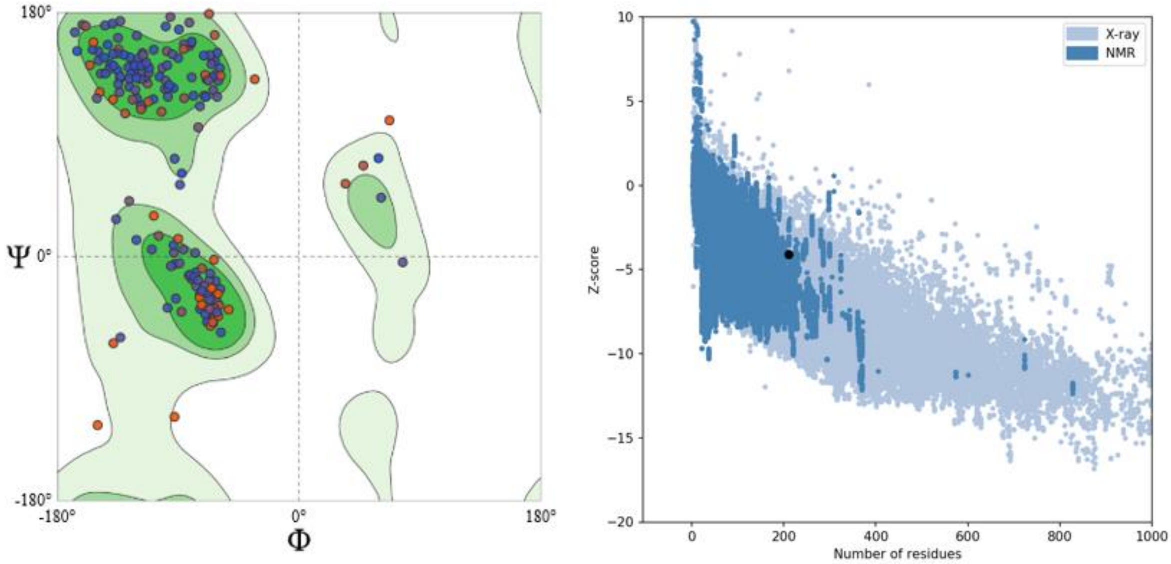

Supplementary Figure S12 Estimating the 3D model quality of consensus VP1 of SA-2018 lineage by Ramachandran plot and Z-score. In the plot, 92.79% of the amino acid residues were in the favored region with 0.96% outliers and Z-score was -4.05.

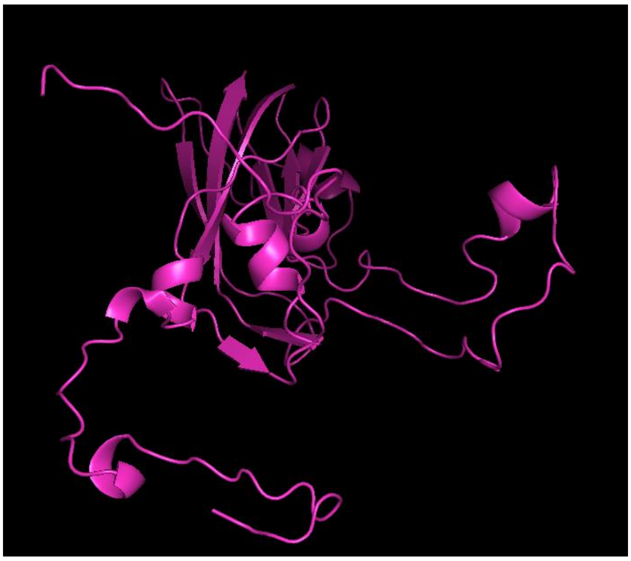

Supplementary Figure S13 3D model of consensus VP1 of SA-2018 isolate generated using PyMOL[11].

Uncategorized Bangladeshi isolates:

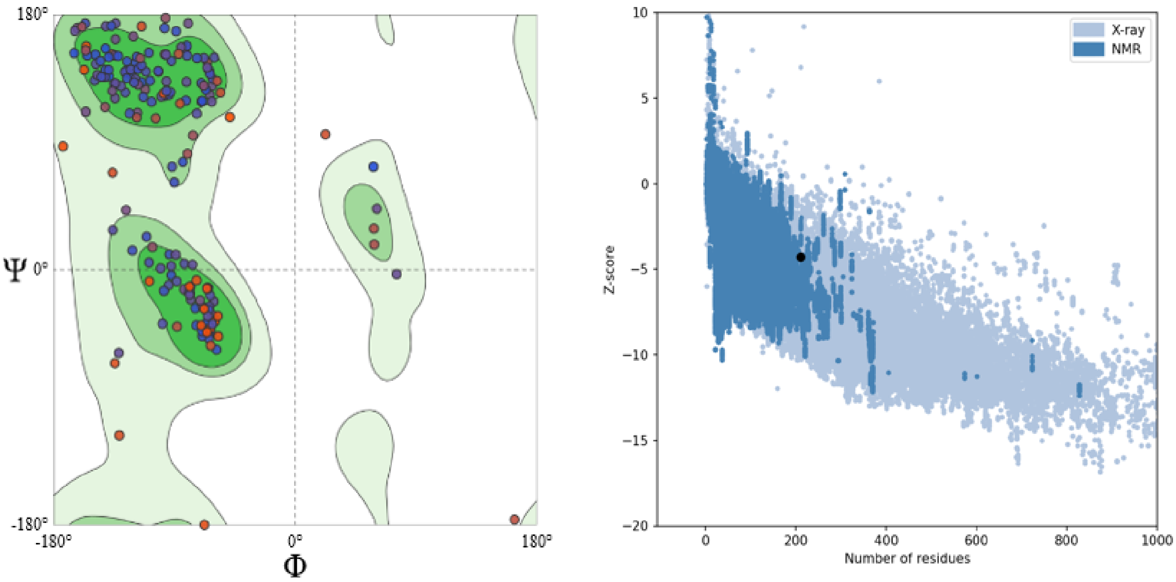

Supplementary Figure S14 Estimating the 3D model quality of consensus VP1 of uncategorized isolates by Ramachandran plot and Z-score. In the plot, 92.23% of the amino acid residues were in the favored region with 1.44% outliers and Z-score was -4.29.

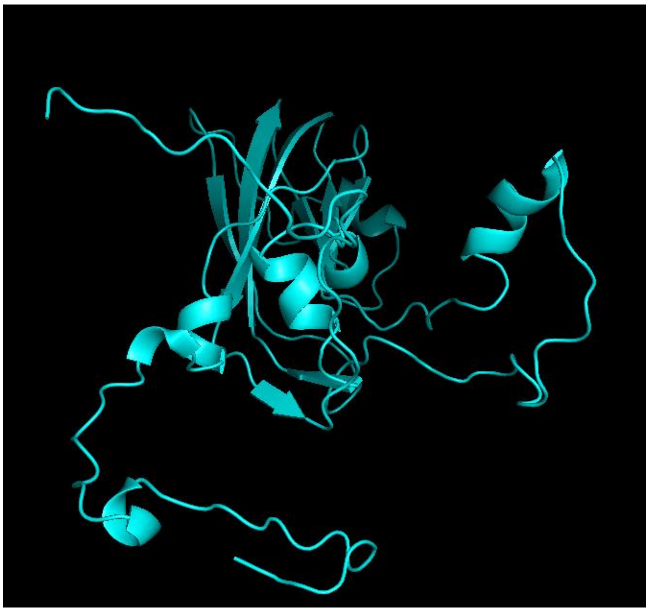

Supplementary Figure S15 3D model of consensus VP1 of uncategorized Bangladeshi isolate generated using PyMOL [11].

Current field vaccine strain (O/India/R2/75):

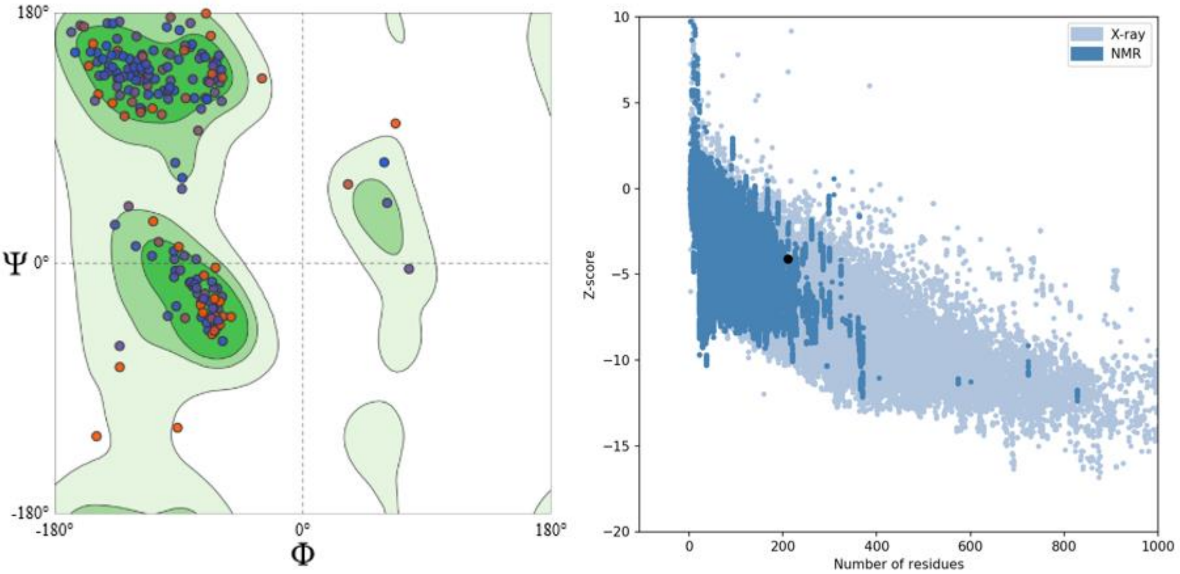

Supplementary Figure S16 Estimating the 3D model quality of VP1 of the current field vaccine strain (O/India/R2/75) by Ramachandran plot and Z-score. In the plot, 93.75% of the amino acid residues were in the favored region with 1.44% outliers and Z-score was -4.1.

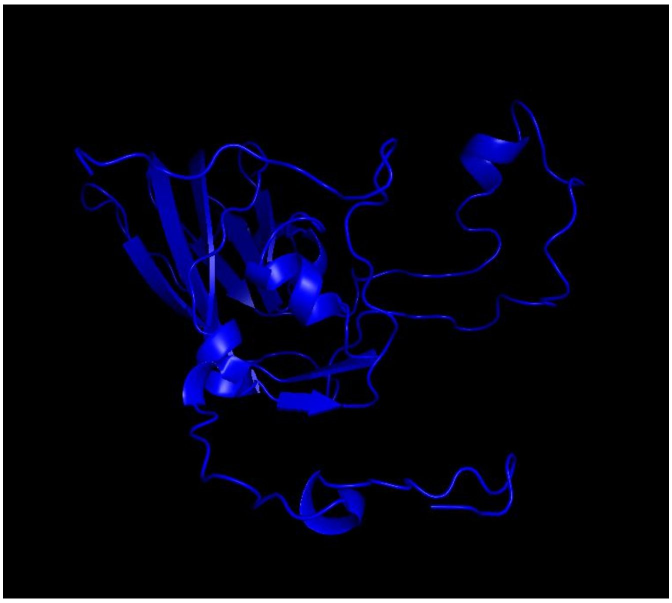

Supplementary Figure S17 3D model of VP1 of the current field vaccine strain (O/India/R2/75) generated using PyMOL [11].

Local vaccine strain (BAN/TA/Dh-301/2016):

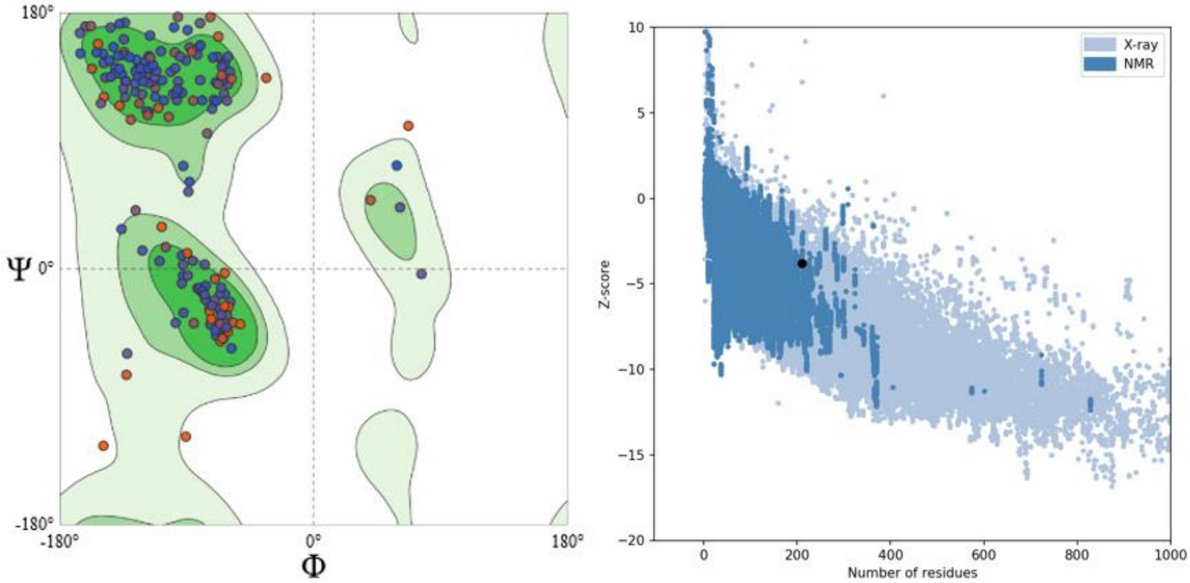

Supplementary Figure S18 Estimating the 3D model quality of VP1 of proposed local vaccine strain (BAN/TA/Dh-301/2016) by Ramachandran plot and Z-score. In the plot, 93.75% of the amino acid residues were in the favored region with 0.48% outliers and Z-score was -3.81.

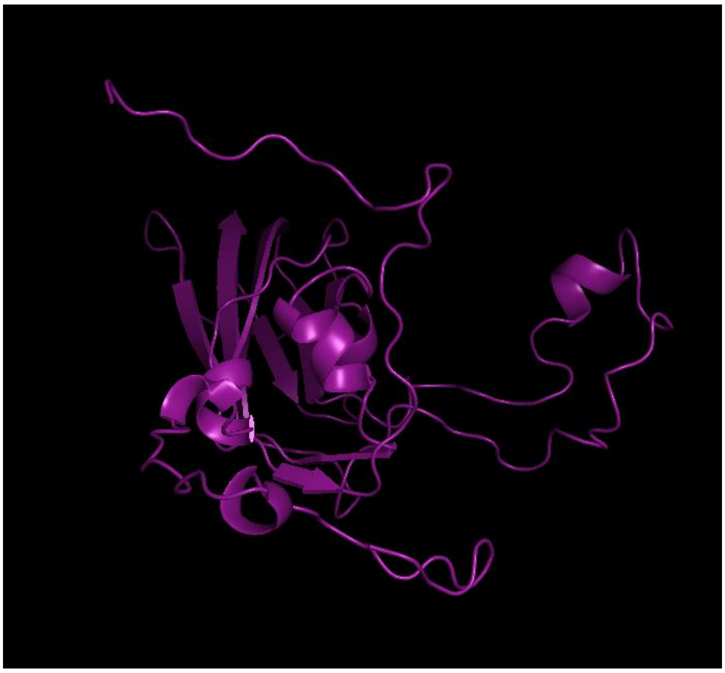

Supplementary Figure S19 3D model of VP1 of proposed local vaccine strain (BAN/TA/Dh-301/2016) generated using PyMOL [11].

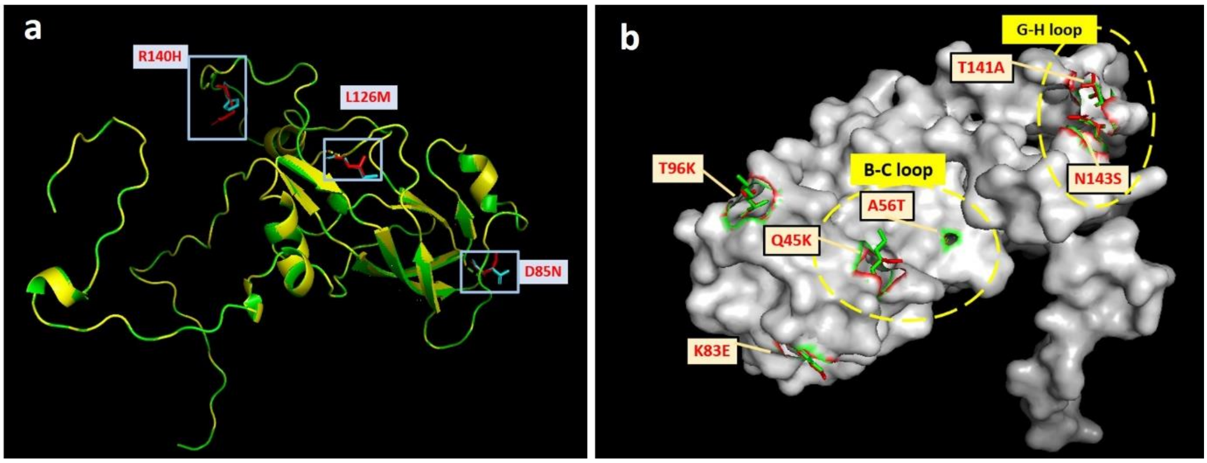

Figure S20 Superimposed three-dimensional (3D) structure of VP1. **(a)** The 3D structure of superimposed MYMBD21 (colored in yellow) and SA-2018 (colored in green) consensus VP1. Unique amino acid substitution sites are represented with stick style (cyan color indicates MYMBD21 amino acids and red color indicates SA-2018 amino acids). **(b)** The 3D structure of consensus VP1 of superimposed MYMBD21 (colored in yellow) and uncharacterized Bangladeshi isolates (colored in green). Unique amino acid substitution sites are represented with stick style (green color indicates MYMBD21 amino acids and red color indicates uncharacterized Bangladeshi isolates' amino acids). Structures were visualized using PyMOL software [11].

2/27/2019 FMDV KIT.jpg

Professor Dr. M. Imdadul Hoque
Dean
Faculty of Biological Sciences
The University of Dhaka
Dhaka-1000, Bangladesh

***Tel:** 58613243, 9673387 (Office)*
***PABX:** 9661900-73/4355, 7545*
***Fax:** (+880-2)-8615583*
***E-mail:** mimdadul07@yahoo.com*
deanbio@du.ac.bd

Ref. ...66.../Biol.Scs./2018-2019 **Date: 14.11.2018**
৩০ কার্তিক, ১৪২৫

Ethical Review Committee

Dr. Munawar Sultana
Associate Professor
Department of Microbiology
University of Dhaka
Dhaka-1000, Bangladesh

Sub: Ethical Clearance.

Dear Munawar Sultana,

With reference to your application on the above subject, this is to inform you that your research proposal entitled **"Development of a simple low-cost diagnostic kit for foot and mouth disease virus"** has been reviewed and approved by the Ethical Review Committee of the Faculty of Biological Sciences, University of Dhaka.

I wish for the success of your research project.

Professor Dr. M. Imdadul Hoque
Dean, Faculty of Biological Sciences
University of Dhaka
Dhaka-1000.

Supplementary Figure S21 Ethical Clearance for the experiment (Ref:66/Biol. Sci./2018-19; Date: 14-11-2018).

References:

1. Tamura, K., Stecher, G. & Kumar, S. MEGA11: Molecular Evolutionary Genetics Analysis Version 11. *Molecular Biology and Evolution* **38**, 3022–3027 (2021).
2. Kitson, J. D. A., McCahon, D. & Belsham, G. J. Sequence analysis of monoclonal antibody resistant mutants of type O foot and mouth disease virus: Evidence for the involvement of the three surface exposed capsid proteins in four antigenic sites. *Virology* **179**, 26–34 (1990).
3. Mateu, M. G. Antibody recognition of picornaviruses and escape from neutralization: a structural view. *Virus Research* **38**, 1–24 (1995).
4. Logan, D. *et al.* Structure of a major immunogenic site on foot-and-mouth disease virus. *Nature* **362**, 566–568 (1993).
5. Strohmaier, K., Franze, R. & Adam, K.-H. Location and Characterization of the Antigenic Portion of the FMDV Immunizing Protein. *Journal of General Virology* **59**, 295–306 (1982).
6. Momtaz, S., Rahman, A., Sultana, M. & Hossain, M. A. Evolutionary Analysis and Prediction of Peptide Vaccine Candidates for Foot-and-Mouth-Disease Virus Types A and O in Bangladesh. *Evol Bioinform Online* **10**, 187–196 (2014).
7. Needleman, S. B. & Wunsch, C. D. A general method applicable to the search for similarities in the amino acid sequence of two proteins. *Journal of Molecular Biology* **48**, 443–453 (1970).
8. Hall, T. A. BIOEDIT: A USER-FRIENDLY BIOLOGICAL SEQUENCE ALIGNMENT EDITOR AND ANALYSIS PROGRAM FOR WINDOWS 95/98/ NT. in (1999).
9. Chen, V. B. *et al. MolProbity* : all-atom structure validation for macromolecular crystallography. *Acta Crystallogr D Biol Crystallogr* **66**, 12–21 (2010).
10. Wiederstein, M. & Sippl, M. J. ProSA-web: interactive web service for the recognition of errors in three-dimensional structures of proteins. *Nucleic Acids Research* **35**, W407–W410 (2007).
11. Schrödinger, LLC. The PyMOL Molecular Graphics System, Version 1.8. (2015).
